# *Mettl3*‐Mediated m6A Modification Represents a Novel Therapeutic Target for FSGS

**DOI:** 10.1002/advs.202501242

**Published:** 2025-12-19

**Authors:** Fubin Zhu, Hongzhi Li, Xiang Li, Chunxiu Du, Ting Wang, Xuling Zhou, Xiaobei Xie, Yunxia Shao, Yingzhi Huang, Yanling Hu, Xinxin Guo, Bin Zhu, Shanshan Yu, Xiaoyan Zhang, Binghai Zhao

**Affiliations:** ^1^ Nephrosis Precision Medicine Innovation Center School of Basic Medicine, Beihua University Jilin 132011 China; ^2^ Health Science Center East China Normal University Shanghai 200241 China; ^3^ Department of Nephrology Wuhu Hospital East China Normal University Wuhu China; ^4^ Department of Nephrology Zhejiang Provincial People's Hospital the Affiliated People's Hospital School of Basic Medicine Hangzhou Medical College Hangzhou Zhejiang 311399 China

**Keywords:** Epegenetics, FSGS, m6A, Mettl3, Tight Junction

## Abstract

Focal segmental glomerulosclerosis (FSGS) is a common glomerular lesion that manifests as a primary podocyte injury. Multiple genetic risk factors have been reported to be associated with the development of FSGS. However, whether epigenetic factors, especially N6‐methyladenosine (m6A) modifications, are involved in the pathogenesis of FSGS remains unclear. By generating a mouse line with a specific deletion of N6‐adenosine‐methyltransferase‐like 3 (*Mettl3*) in podocytes (*Mettl3* ^podko^mice), podocytes are isolated and performed RNA‐seq. It is shown that RNA m6A methylation levels are reduced in the renal glomeruli of both animal models and patients with FSGS. A significant decrease in RNA m6A levels in podocytes and the development of an FSGS phenotype are observed in the *Mettl3* ^podko^ mice. Furthermore, RNA‐seq and m6A‐immunoprecipitated RNA sequencing revealed that silencing *Mettl3* expression in podocytes led to a gene expression profile associated with slit diaphragm dysfunction. RNA immunoprecipitation assay and hybridization chain reaction (HCR) analysis further identified the slit diaphragm marker TJP1 as a potential target of Mettl3. Moreover, loss‐ and gain‐of‐function analyses showed that Mettl3 enhances podocyte RNA m6A modification, probably through the TJP1–CDC42 pathway. Finally, treatment of *Mettl3* ^podko^ and adriamycin (ADR)‐induced FSGS mice with m6A‐mimic compounds markedly ameliorated the progression of FSGS. The findings demonstrate that *Mettl3*‐mediated RNA m6A modification is essential for maintaining podocyte architecture and function and represents a potential therapeutic target for FSGS.

## Graphical abstract

This study explores the roles of Mettl3‐induced N6‐methyladenosine (m6A) modifications in Focal segmental glomerulosclerosis (FSGS). The findings reveal that inhibition of Mettl3 results in podocyte injury by modulating the TJP1CDC42 pathway. Moreover, Administration of N6‐methyladenosine attenuates the FSGS phenotype in WT mice induced by Adriamycin and Mettl3 deficiency.

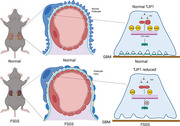

## 1 Introduction

Focal segmental glomerulosclerosis (FSGS) is a histopathological lesion characterized by varying degrees of podocyte foot process effacement and obliteration of the capillary lumen. FSGS mainly originates from direct podocyte injury and is the most likely to progress to end‐stage renal disease (ESRD) among primary nephrotic diseases.^[^ ^1^, ^2^ ^]^ FSGS can occur at any age and accounts for ≈20% of nephrotic syndrome cases in children and 40% in adults.^[^ ^3^, ^4^ ^]^ FSGS has diverse etiologies, including mutations in key podocyte genes, circulating factors (soluble urokinase‐type plasminogen activator receptor (suPAR), infections, and drug use (e.g., heroin).^[^ ^5^ ^]^ However, due to its unclear pathogenesis, there is currently no specific treatment for FSGS.

Podocytes are terminally differentiated cells that play a crucial role in the renal glomerular filtration barrier through their foot processes. Podocyte foot processes form complex interdigitations with adjacent foot processes of neighboring podocytes. Multiple interdigitating foot processes are interconnected by slit diaphragms that span the surface of the glomerular basement membrane (GBM) and form the final barrier to filtration. Dysfunction of podocyte‐specific genes can disrupt the GBM structure, resulting in the effacement of foot processes and detachment of podocytes. Several genes selectively expressed in podocytes have been reported to be associated with FSGS, such as the slit diaphragm gene NPHS1, tight junction protein 1 (TJP1), and CD2‐associated protein (CD2AP).^[^ ^6^, ^7^, ^8^, ^9^ ^]^ Mutations in these genes can disrupt the slit diaphragm complex and cause proteinuria, a hallmark of FSGS. In addition, nuclear transcription factors, including WT1 and LMX1B, are essential for podocyte development and maintenance, and mutations in these factors have been reported to be involved in FSGS.^[^ ^10^, ^11^, ^12^ ^]^

RNA contains more than 100 modifications. Among these, N6‐methyladenosine (m6A) methylation is considered the most common, abundant, and conserved modification, particularly in eukaryotic messenger RNAs (mRNA). m6A methylation is a key determinant of the post‐transcriptional regulation of mRNA splicing, translation, and degradation. It is catalyzed by multiple methyltransferases, including methyltransferase‐like 3 and 14 (METTL3/14) and Wilms’ tumor 1‐associating protein (WTAP), referred to as “writers,” and is demethylated by ALKBH5 and FTO, termed as “erasers”.^[^ ^13^ ^]^ m6A methylation is involved in many biological processes, including embryonic stem cell development,^[^ ^14^ ^]^ spermatogenesis,^[^ ^15^ ^]^ cancer progression,^[^ ^16^ ^]^ and cardiac hypertrophy.^[^ ^17^ ^]^ Recent reports have shown that *Mettl3*‐mediated m6A methylation is associated with the pathogenesis of polycystic kidney disease and diabetic nephropathy (DN).^[^ ^18^, ^19^ ^]^ Another report also showed elevated m6A levels and Mettl14 expression in patients with FSGS and DN and animal models, respectively.^[^ ^20^ ^]^ However, the role and mechanism of m6A modification in kidney diseases, such as FSGS, remain unclear.

In the present study, we report that m6A modification and *Mettl3* expression in the renal glomeruli were decreased in mouse models and patients with FSGS. We showed that specific deletion of *Mettl3* in mouse podocytes (*Mettl3* ^podKO^) resulted in an FSGS‐like phenotype. Enhancing m6A RNA methylation by treating the mice with N6‐methyladenosine markedly ameliorated FSGS progression in the *Mettl3* ^podKO^ mice and attenuated adriamycin (ADR)‐induced FSGS.

## 2 Experimental Section

### 2.1 Patient Samples

This study was approved by the Ethics Review Board of Jilin City Central Hospital, Jilin (Approval Number: BHJL: NO.2022‐05). Formalin‐fixed paraffin‐embedded (FFPE) and frozen renal biopsy samples (n = 14) were obtained with written informed consent from the Jilin City Central Hospital Renal Biopsy Archive. Control samples (n = 8) were obtained from paracancerous tissues in patients who underwent surgical removal of renal carcinoma. The clinical characteristics of all patients included in this study are detailed in Table S1 (Supporting Information). In all cases, no patient received immunosuppressive therapy at the time of kidney biopsy.

### 2.2 Animal Treatment

Conditional *Mettl3* ^flox/flox^ mice were originally created by BRL Medicine Inc. (Shanghai, China) and were kindly provided by Dr. Fei Sun, Zhejiang University. Podocyte‐specific *Mettl3* knockout mice (*Mettl3* ^podko^) were generated by crossing *Mettl3* floxed mice with Nphs2‐Cre transgenic mice (Cyagen, Suzhou, China), and *Mettl3* ^podko^ mice were confirmed by genotyping (Figure S2A, Supporting Information). Age‐matched male *Mettl3* ^podko^ mice were used, and male Nphs2‐Cre mice served as wild type (WT). N6‐methyladenosine (2 mg kg^−1^ on alternate days; Aladdin Shanghai, China) and glucocorticoids (GC, 1 mg kg^−1^ day^−1^) were intraperitoneally injected into *Mettl3* ^podko^ mice from 5 to 7 months of age (Figure 5B). In another FSGS‐prone animal model, ADR‐treated 8‐week‐old male BALB/c mice (Charles River, Beijing, China) were housed under standardized conditions for 2 weeks and then received a single tail vein injection of ADR (Doxorubicin HCl; Solarbio, Beijing, China) at a dose of 10 mg kg^−1^ (n = 10). N6‐methyladenosine (2 mg kg^−1^ on alternate days; n = 10), or GC (1 mg kg^−1^ day^−1^; n = 10) was administered via intraperitoneal injection (Figure 6A). All animal experiments were conducted in accordance with protocols approved by the Ethics Committee of Laboratory Animal Care and Welfare, Hangzhou Medical College (Approval Number: ZJCLA‐IACUC‐20040082). All mice were housed in sterile individually ventilated cages under specific pathogen‐free conditions and provided with water and sterile growth and reproductive food (KeAoXieLi Feed Co., Ltd., Beijing, China; SPF). Male littermate mice of the same age were used in this study, unless otherwise stated. Following collection, kidney, spleen, and gut specimens, as well as serum, were stored at −80 °C.

### 2.3 RNA‐Sequencing

Using isolated murine podocytes from WT (n = 3) and *Mettl3* ^podko^ (n = 3) mice, all large and small RNAs were isolated using the mirPremier microRNA Isolation Kit (Ambion Inc., USA). mRNA libraries were constructed using the TruSeq Stranded Total RNA Library Prep Kit (NEB Inc., Ipswich, MA, USA). Sequencing was performed on a HiSeq 2000 sequencer with PE50 at BGI (Shenzhen, China). The isolated podocyte RNA‐seq data were available in the SRA under accession number PRJNA1017102.

### 2.4 Methylated RNA Immunoprecipitation Sequencing

MeRIP‐seq was performed according to a previously reported protocol. In brief, total RNA isolated from the podocyte of the WT and *Mettl3* ^podko^ mice using mirPremier microRNA Isolation Kit (Ambion Inc., USA), and 200 ng of RNA was fragmented with RNA Fragmentation Reagents (AM8740, Invitrogen) into ≈100‐nucleotide fragments. Dynabeads Protein G and SuperBlock T20 (Life Technologies) were mixed with fragmented RNA at 4 °C for at least 2 h with rotation. Fragmented RNA was incubated with anti‐m6A antibody at 4 °C overnight in IP buffer [150 mm NaCl, 10 mM Tris‐HCl, and 0.1% NP‐40 supplemented with RNase and protease inhibitors]. The bead–antibody–RNA complexes were washed twice with IP wash buffer. After a final wash, proteinase K was added, and the mixture was incubated at room temperature for 1 h. Both input and m6A‐IP pulldown RNAs were prepared for next‐generation sequencing (BGI, Shenzhen, China), similar to regular RNA‐seq. The isolated podocyte m6A RNA‐seq data for 5‐month‐old male WT and *Mettl3* ^podko^ mice were available in the SRA under accession number PRJNA1017102. All m6A‐seq results are provided in the Supporting Information.

### 2.5 RNA Immunoprecipitation

RIP was performed using the Magna RIP RNA‐Binding Protein Immunoprecipitation Kit (Millipore) according to the manufacturer's instructions. Briefly, MPC cells transfected with GFP‐*Mettl3* lentivirus were lysed in complete RIP lysis buffer (1 × 10^7^ cells). Magnetic beads coated with 5 µg of specific antibodies against mouse IgG (Millipore) or GFP (Proteintech, Wuhan, China) were incubated with prepared cell lysates at room temperature for 4 h. The RNA–protein complexes were then washed six times and incubated with proteinase K digestion buffer to isolate the immunoprecipitated RNA. RNA was collected and reverse‐transcribed into cDNA, and TJP1 and other genes were quantified by qPCR.

### 2.6 RNA‐Seq Bioinformatics Analysis

The sequencing data were filtered using SOAPnuke^[^ ^21^ ^]^ (v2.2.1), and the clean reads were mapped to the reference genome with HISAT2^[^ ^22^ ^]^ (v2.2.1), then assigned to gene regions in GTF files (mm9 genome assembly) using Bowtie2^[^ ^23^ ^]^ (v2.4.5). Gene expression levels were calculated using RSEM^[^ ^24^ ^]^ (v1.3.1). Differential expression analysis was performed using DESeq2^[^ ^25^ ^]^ (v1.34.0) with Q ≤ 0.05 and |log2 fold change| > 1. KDA analysis was conducted in accordance with previously reported protocols.^[^ ^26^ ^]^

### 2.7 Hybridization Chain Reaction (HCR)

On day 1, the MPC5 cells were fixed with 4% paraformaldehyde and washed with PBST five times for 5 min. Incubated the MPC 5 cells for 10 mins with PBS containing 0.25% Triton X‐100 and washed the cells in PBS three times for 5 min. Blocked cells with 1% BSA in PBST for 1 h and subsequently incubated with METTL3 antibody diluted in 1% BSA in PBST overnight at 4 °C. Hybridization buffer (50% formamide, 5 × sodium chloride–sodium citrate, 9 mM citric acid pH = 6.0, 0.1% Tween‐20, 50 µg mL^−1^ heparin, 1 × Denhardt's solution, 10% dextran sulfate) and primers were added (primers are listed in Table S5, Supporting Information), and cells were incubated at 37 °C overnight. On day 2, cells were washed with 300 µL probe wash buffer (50% formamide, 5 × sodium chloride–sodium citrate, 9 mm citric acid, pH 6.0, 0.1% Tween‐20, 50 µg mL^−1^ heparin) three times for 15 min at 37 °C, followed by washing with 1 mL 5 × sodium chloride–sodium citrate and 0.1% Tween‐20 at room temperature 3 times for 15 min. Amplification was performed by incubating samples with amplification buffer (5 × sodium chloride–sodium citrate, 0.1% Tween‐20, 10% dextran sulfate) and amplifier (Sangon Biotech, China) at room temperature overnight. On day 3, cells were washed with 1 mL 5 × SSCT (0.75 M NaCl, 0.075 M sodium citrate, and 0.1% Tween‐20) twice for 5 min, followed by washing with 1 mL 5 × SSCT for 30 min twice, and finally washed with 1 mL 5 × SSCT for 5 min. The samples were imaged using a Nikon A1 Ti system. All assays were repeated at least three times.

### 2.8 ELISA and Urine Analysis

ELISA for the total RNA m6A (Abcam, Shanghai, USA) content of 500 ng of RNA was performed according to the manufacturer's instructions. For urine analysis, urine was collected from mice overnight using metabolic cages and stored in −80°C. ELISA for murine urine albumin (Assaypro, Charles, USA) was performed as previously described. Urinary creatinine was measured using a creatinine kit (Nanjing Jiancheng Bioengineering Institute, Nanjing, China), and proteinuria was expressed as milligrams of albumin per millimole of creatinine.

### 2.9 Cell Culture and Treatments

Mouse podocytes (MPC) were kindly provided by Professor Xujie Zhou of Peking University. The MPC cells were cultured under growth‐permissive conditions at 33 °C in RPMI 1640 supplemented with 10% fetal bovine serum (FBS), 20 U mL^−1^ mouse recombinant interferon‐γ (IFN‐γ), and 100 U mL^−1^ penicillin plus 0.1 mg mL^−1^ streptomycin. To induce differentiation, podocytes were maintained in non‐permissive conditions at 37 °C without IFN‐γ for 7 days and used for the experiments. Transfection of MPC cells was performed for 72 h using Lipofectamine 2000 (Invitrogen Life Technologies, Carlsbad, CA, USA) according to the manufacturer's instructions with *Mettl3* siRNA (100 nmol L^−1^; OBIO Technology, Shanghai, China), *Cdc42*, and *Tjp1* siRNA (100 nmol L^−1^; Genepharma, Shanghai, China), and negative control siRNA (100 nmol L^−1^; OBIO Technology, Shanghai, China). For *Mettl3* overexpression, GFP and a *Mettl3* lentivirus vector (OBIO Technology, Shanghai, China) at a dose of 40 Multiplicity of Infection (MOI) were added to MPC cells directly, and scrambled controls were used in parallel. To mimic FSGS phenotype in vitro, MPC cells were treated with ADR at a final concentration of 0.1 µg mL^−1^ in culture medium.

### 2.10 Cell Counting Kit‐8 Assay

The MPC cells were seeded in 96‐well plates at a density of 5 × 10^3^ cells/well. The next day, the cells were transfected with *Mettl3* siRNA or negative siRNA (100nM L^−1^) for 24 h. The medium was replaced with serum‐free medium containing 10% Cell Counting Kit‐8 (CCK‐8) reagent (Biosharp Life Sciences, Anhui, China) after 1 h of incubation. The absorbance was measured at 450 nm using a plate reader (Infinite M200 PRO, Switzerland).

### 2.11 RNA Stability

The MPC cells were seeded in six‐well plates with 2 × 10^5^ cells in each well and were incubated with actinomycin (5 µg/ml) to terminate transcription. Samples were collected at 0, 3, and 6 h after termination. Total RNA was extracted, and *TJP1* mRNA expression was determined by real‐time PCR.

### 2.12 Glomeruli Isolation and Podocyte Culture

For isolation of the glomeruli, the kidneys from WT (n = 3) and *Mettl3* ^podKO^ mice (n = 3) were cut into small pieces with a scalpel in 3 ml HBSS and treated with collagenase II (2 mg/ml, Biofroxx, Guangdong, China) in HBSS at 37 °C and shaking for 1 h. The enzyme‐digested kidney tissue was transferred onto a 100‐mm tissue strainer and washed with 30 mL HBSS. Subsequently, the suspension was passed through a 70 mm sieve and rinsed with an additional 15 mL PBS. PBS containing the glomeruli and renal tubules was passed through a 40 mm sieve, and the procedure was repeated. The glomeruli retained on the sieve were back‐flushed with a pipette into a new 15 mL tube, and the collected glomeruli fraction was centrifuged for 10 min at 2000 rpm. The glomeruli were collected and transferred to a cell‐culture dish, and cultured for 7 days at 37 °C in RPMI 1640 supplemented with 10% fetal bovine serum (FBS), and 100 U mL^−1^ penicillin plus 0.1 mg mL^−1^ streptomycin.

### 2.13 Histological Analysis of the Kidney

For immunofluorescence and immunohistochemistry assays, paraffin embedded kidney sections (3 µm) were incubated with antibodies against m6A (Abcam, Shanghai, USA), Wtap (SantaCruz, Shanghai, USA), Mettl3 (Invitrogen, Shanghai, USA), Mettl14/Podxl/Nephrin (Bioss Antibodies, Beijing, China), Tjp1/Cldn1/Cdc42/Wasp/Fn (Proteintech, Wuhan, China), α‐SMA (Abcam, Shanghai, USA). For hematoxylin and eosin (H&E) and Periodic acid–Schiff (PAS) staining, kidney sections (3 µm) were stained with H&E and PAS (Solarbio) according to the manufacturer's protocols. Detailed antibody information is provided in Table S4 (Supporting Information).

### 2.14 Western Blotting and Immunoprecipitation

Kidney tissues and MPC cells were homogenized in lysis buffer (Thermo Fisher Scientific, Shanghai, USA) containing 0.025 M Tris, 0.15 M NaCl, 0.001 M EDTA, 1% NP‐40, 5% glycerol, pH 7.4, and a protease inhibitor (Roche Hong Kong Limited, Hong Kong, China). The total protein in each sample was quantified using the BCA assay kit (Thermo Scientific, IL, USA). Samples (10 µg/lane) were resolved by SDS‐PAGE, western blotted (WB), and probed with the following primary antibodies: anti‐Mettl3/Tjp1/Cdc42/Wasp (Proteintech, Wuhan, China). β‐actin (ZSGB‐BIO, Beijing, China) staining was used as a loading control. Antibody information is presented in Table S4 (Supporting Information).

### 2.15 Ultrastructural Analysis

Kidney tissues were fixed in 2.5% glutaraldehyde in 0.1 M phosphate buffer (pH 7.4) at 4 °C for 24 h. The samples were then washed with phosphate buffer (0.1 M, pH 7.4) for 12 h and post‐fixed for 20 min in 1% OsO4 in 0.1 M phosphate buffer (pH 7.4). The samples were then washed with phosphate buffer (0.1 M, pH 7.4) for 30 min, dehydrated, and embedded in Epon. Thin sections (50 nm) were placed on copper grids and stained with 2% uranyl acetate and 1% lead citrate for 30 min. A JEM‐1010 transmission electron microscope was used to visualize the ultrastructures. Ten randomly selected areas from each specimen were photographed and analyzed using the Image ProPlus software (Image‐Pro Plus, Media Cybernetics, USA).

### 2.16 RNA Extraction and Analysis

RNA was extracted from the kidney tissue and podocytes using an miRNA isolation kit (Ambion Inc., Austin, TX, USA) to separate large and small RNAs according to the manufacturer's instructions. RT‐qPCR was performed using a final reaction volume of 20 µL containing 9 µL Fast Start Universal SYBR Green Master Mix (Roche, Shanghai, China), 7.4 µL nuclease‐free water, 0.8 µL mRNA primers, and 2 µL RT product. Data were normalized to RPS16 (ribosomal protein S16) using a standard curve method to account for differences in reverse transcription efficiencies and template amounts in the reaction mixtures. All primer sequences are listed in Table S2 (Supporting Information).

### 2.17 Luciferase Assay

*TJP1*and other genes, a gene luciferase vector containing *Mettl3* response elements was amplified from mouse cDNA by PCR. Plasmid DNA and *Mettl3* overexpression vector were co‐transfected into HEK293A cells for 48 h. Luciferase activity was measured using a plate reader (Infinite M200 PRO, Switzerland) and normalized by measuring β‐galactosidase activity. The primers used to generate specific fragments of mouse *TJP1*‐related gene sequences are listed in Table S3 (Supporting Information).

### 2.18 Statistical Analyses

Data were expressed as mean ± SD and compared using one‐way ANOVA and t tests with Dunnett's Multiple Comparison in Prism (version 8; GraphPad Software, La Jolla, CA). Statistical significance was set at *p* < 0.05.

## 3 Results

### 3.1 N6‐Methyladenosine Levels are Decreased in the Glomeruli of Mice and Patients with FSGS

**Figure 1:**
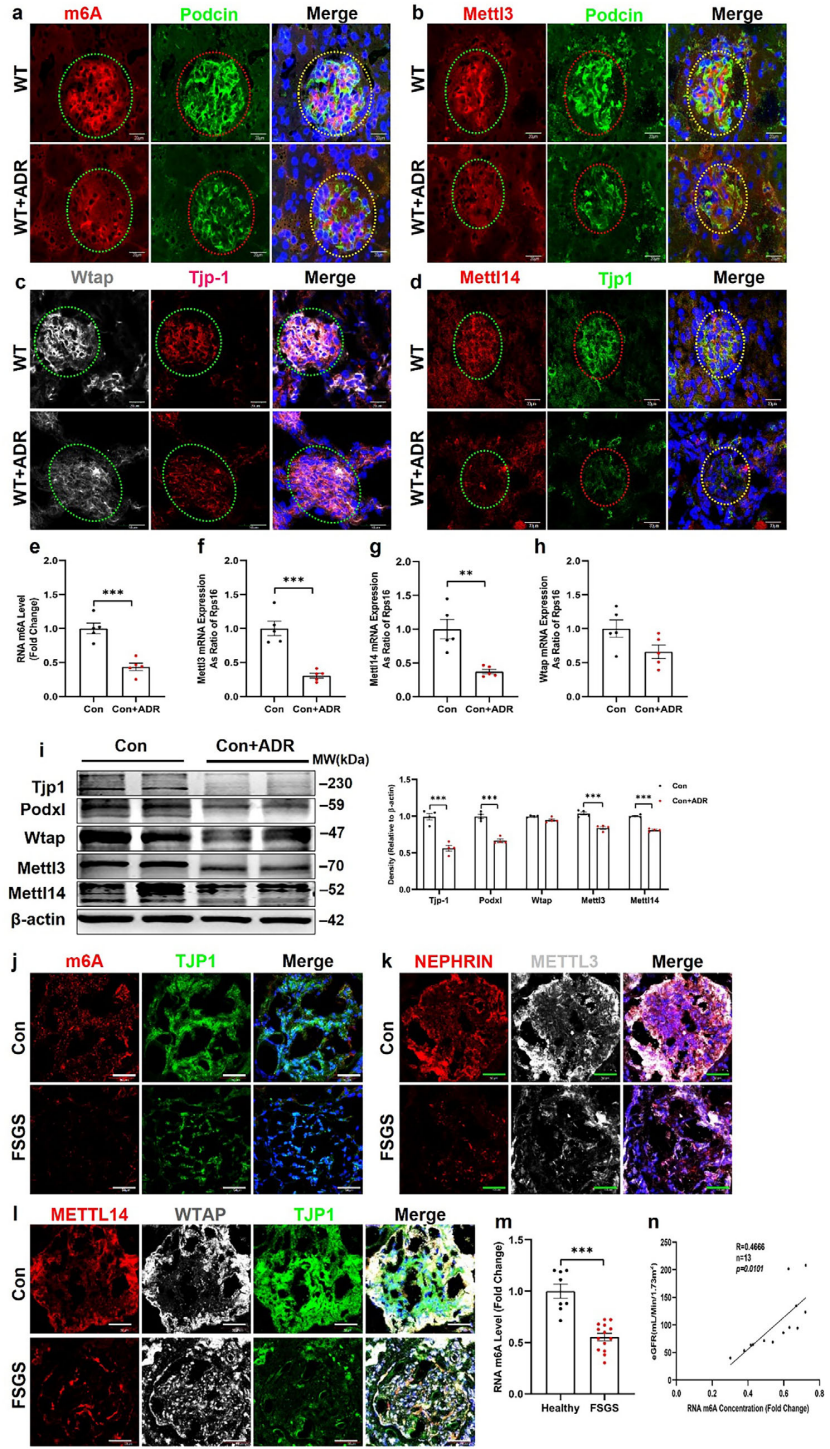
The RNA m6A levels and Mettl3/14 expression are decreased in glomeruli of adrimycin‐treated mice podocytes. a,b) Representative images of m6A and its catalyzing enzyme Mettl3 staining in the kidneys of ADR‐treated mice (n = 6, Scale bar = 40 µm). c,d) Representative images of Mettl14 and Wtap staining in kidneys of ADR‐treated mice (n = 6, Scale bar = 40 µm). e–h) Quantification of m6A levels and Mettl3/14 or Wtap expression in MPC cells treated with or without ADR using ELISA and qPCR, respectively (n = 5). i) Representative western blot analysis of Tjp1, Podxl, Mettl3/14, and Wtap in MPC cells treated with ADR, the right panel shows quantification (n = 3). j,k) Representative images of m6A and its catalyzing enzyme, Mettl3, in kidney sections of patients with FSGS (n = 14, Scale bar = 50 µm). l) Representative images of Mettl14 and Wtap staining in kidney sections from a patient with FSGS (n = 14, Scale bar = 50 µm). m) Quantification of RNA m6A methylation levels in renal biopsies from Control and FSGS patients (n = 14). n) Correlation between RNA m6A methylation levels and eGFR in patients with FSGS (n = 13). ^**^ *p* < 0.01; ^***^ *p* < 0.001. Data are shown as mean ± SD.

To confirm whether RNA m6A methylation is involved in FSGS, we used mice with an FSGS phenotype induced by adriamycin (ADR, Figure S1a–c, Supporting Information). We observed significant downregulation of RNA m6A methylation levels and of Mettl3, Mettl14, and Wtap expression in the renal tissues of ADR‐induced FSGS mice compared with that in non‐treated control mice (**Figure** **1**a–d; Figure S1d–i, Supporting Information). Consistently, in cultured mouse podocyte cells (MPCs), ADR treatment led to a significant decrease in RNA m6A methylation levels, as well as in the mRNA and protein expression of Mettl3 and Mettl14 (Figure 1e–i; Figure S1j, k, Supporting Information).

The aim of this study was to further clarify the role of RNA m6A methylation in the pathogenesis of FSGS. We measured m6A levels in renal biopsies from patients with FSGS. Immunofluorescence analysis of RNA m6A methylation demonstrated a significant decrease in the renal glomeruli of patients with FSGS compared with control renal tissues obtained from para‐renal carcinoma (Figure 1j). In line with the changes in RNA m6A methylation, the levels of Mettl3, Mettl14, and Wtap were also reduced in the glomeruli of patients with FSGS (Figure 1k and l; Figure S1l–q, Supporting Information). Similarly, expression of TJP1 in the glomeruli was downregulated in the kidneys of patients with FSGS (Figure 1j,l). Furthermore, RNA ELISA revealed that RNA m6A methylation levels were significantly decreased in kidney biopsies from patients with FSGS compared with control paracancerous tissues (Figure 1m). The extent of RNA m6A modification was significantly correlated with eGFR levels in FSGS (Figure 1n). These findings demonstrate that RNA m6A modification levels are reduced, along with the downregulation of multiple methyltransferases, including Mettl3, Mettl14, and Wtap, in renal glomeruli of mice and patients with FSGS.

### 3.2 Podocyte‐Specific Deletion of Mettl3 Results in FSGS

**Figure 2:**
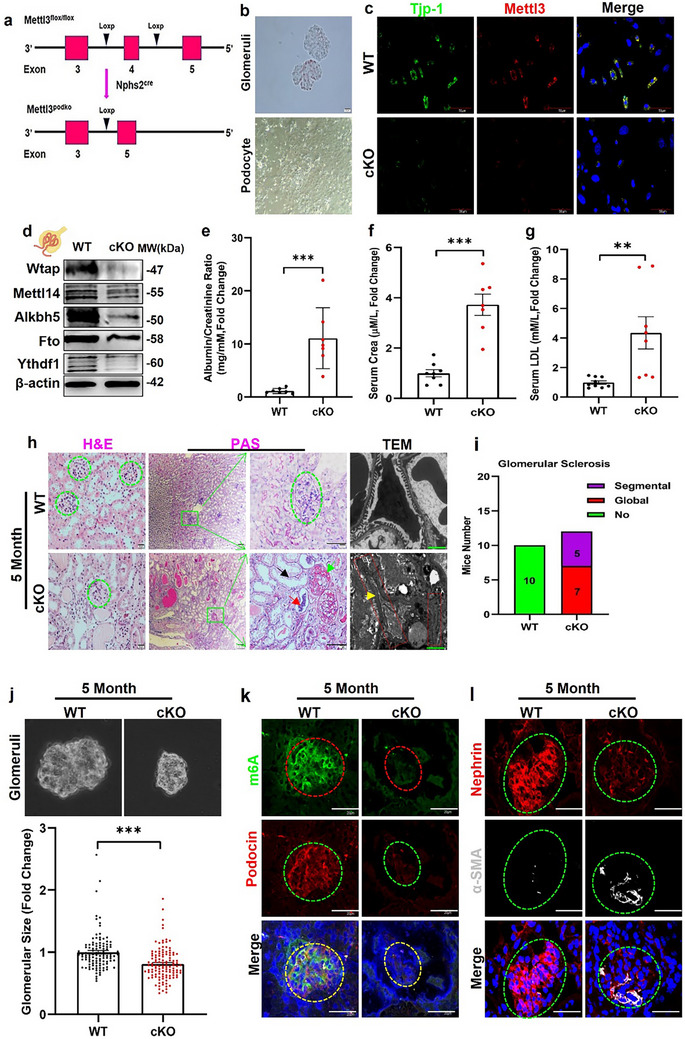
Deletion of *Mettl3* in the podocyte results in FSGS in 5‐month‐old mice. a) Strategy for generating the *Mettl3* ^podKO^ mice: loxP recombination sites were introduced in introns 3 and 5, after which exon 4 was removed. b) Images of isolated glomeruli and cultured podocytes from WT at 5‐month‐old (n = 3). c) Representative immunostaining of Mettl3 and Tjp1 in cultured podocytes from glomeruli isolated from WT and *Mettl3* ^podKO^ mice (n = 3). d) Representative western blot analysis of Wtap, Mettl14, Alkbh5 and Fto, or Ythdf1 in isolated glomeruli (n = 3). e–g) Quantification of proteinuria, serum creatinine, and LDL in 5‐month‐old WT and *Mettl3* ^podKO^ mice (n = 7). h) Representative images of HE (n = 6)and PAS (n = 8, Scale bar = 60 µm) or TEM (n = 6, Scale bar = 2 µm) staining showed protein and cellular casts in tubules (red arrow), tubular dilation (black arrow), severe glomerular damage such as multifocal adhesions broad capsular adhesions, and narrow capillary lumen (green arrow) and foot process effacement (yellow arrow) in 5‐month old *Mettl3* ^podKO^ mice (TEM, Scale bar = 2 µm). i) Quantification of the rate of *Mettl3* ^podKO^ mice with glomerular sclerosis. j) Representative image of glomeruli size isolated from WT (n = 111) and *Mettl3* ^podKO^ (n = 119) mice. k) Representative immunofluorescence images of m6A and the slit diaphragm protein podocin in kidney sections from the WT and *Mettl3* ^podKO^ mice at 5 months old (n = 6, Scale bar = 40 µm). l) Representative images of the slit diaphragm protein nephrin and fibrosis marker α‐SMA staining in kidney sections from *Mettl3* ^podKO^ mice at 5 months old (n = 6, Scale bar = 40 µm). ^**^ *p* < 0.01; ^***^ *p* < 0.001. Data are shown as mean ± SD.

**Figure 3:**
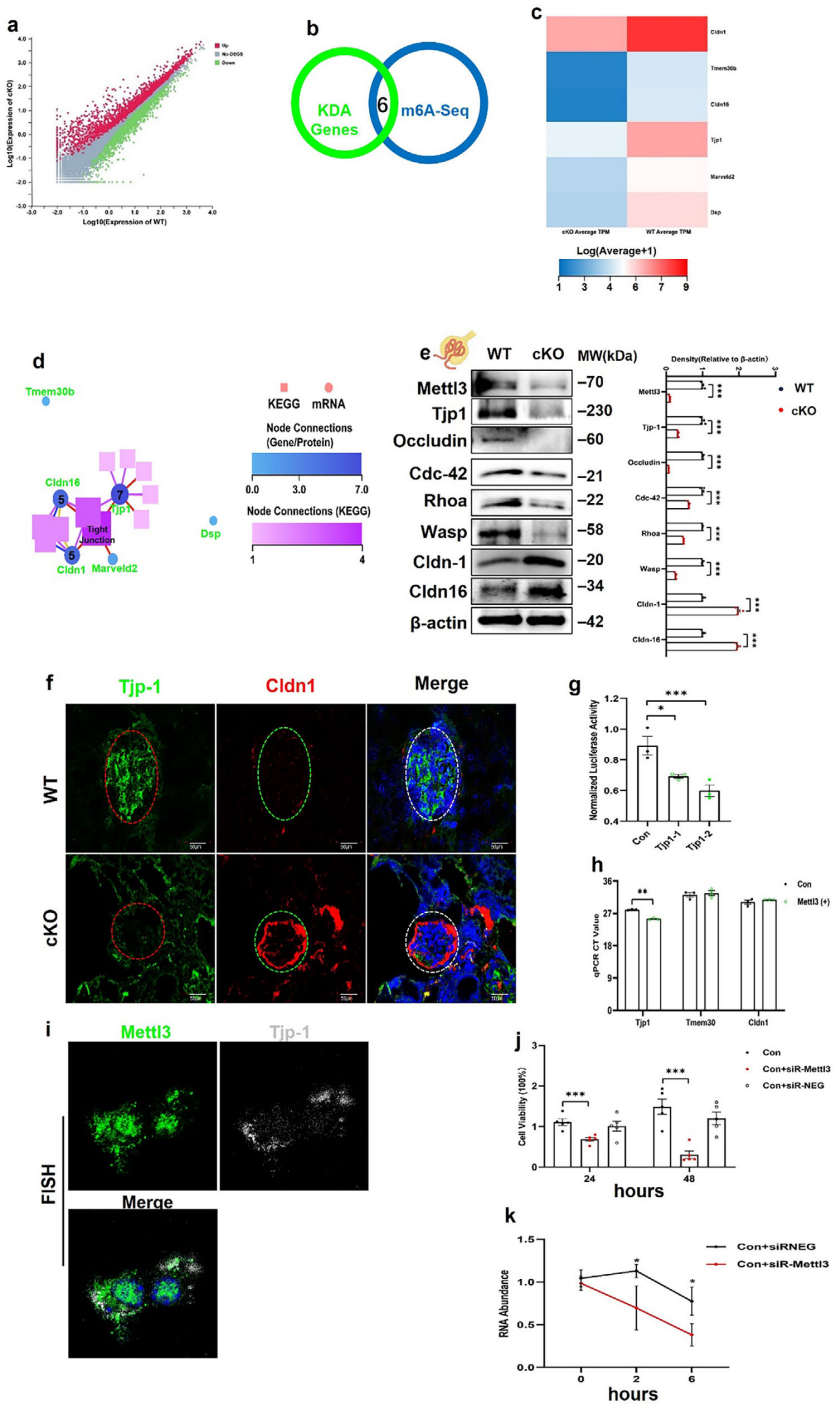
Mettl3 binds to Tjp1 to regulate podocyte slit diaphragms. a) Scatter plot of differentially expressed genes in podocytes cultured from glomeruli isolated from WT and *Mettl3* ^podKO^ mice. b) Venn diagram showing the number of genes in podocytes with significant changes in WT and *Mettl3* ^podKO^ mice and those identified as potential *Mettl3* targets. c) Heatmap of differentially expressed genes from the Venn diagram. d) KEGG pathway relationship network for six genes from the overlapped Venn diagram; Arabic numerals indicate gene node connections. e) Representative western blot analysis of Mettl3, Tjp1, Occludin, Cldn1/16, Cdc42, and Rhoa or Wasp from isolated glomeruli. The right panel shows quantification (n = 4). f,) Representative images of Tjp1 and Cldn1 staining in kidney sections from WT and *Mettl3* ^podKO^ mice at 5‐month‐old (n = 6, Scale bar = 40 µm). g) Luciferase activity in HEK 293A cells transfected with Mettl3 and Tjp1 reporter constructs, demonstrating the binding of Mettl3 to the Tjp1 reporter. h) Quantification of METTL3 RIP pull‐down mRNA expression by qPCR in *Mettl3* lentivirus‐transfected MPCs (n = 3). i) Representative image of hybridization chain reaction (HCR) showing colocalization of *Tjp1* mRNA and Mettl3 in MPC5 cells (n = 6). j) Quantification of cell viability by the CCK8 assay in *Mettl3* siRNA‐transfected MPC cells (n = 5). k) Decay rate of *Tjp1* mRNA after actinomycin D (5 µg mL^−1^) administration in *Mettl3* siRNA‐transfected MPC cells (n = 4). ^*^ *p* < 0.05; ^**^ *p* < 0.01 and ^***^ *p* < 0.001. Data are shown as mean ± SD.

To further clarify the role of RNA m6A methylation in podocytes, we generated a conditional *Mettl3* gene knockout mouse line (*Mettl3* ^flox/flox^) carrying the floxed *Mettl3* allele. By mating with Nphs2‐Cre mice, we generated mice with podocyte‐specific deletion of the *Mettl3* gene and confirmed this by tail DNA genotyping (**Figure** **2**a). Furthermore, immunofluorescence confirmed the absence of Mettl3 and Tjp‐1protein and mRNA expression in primary cultured podocytes from 5‐month‐old mice (Figure 2b, c; **Figure** **3**e; Figure S2a–d, Supporting Information). To determine whether deletion of *Mettl3* led to compensatory effects on other m6A‐related enzymes, especially Mettl14 or Wtap, we observed significant reductions in Mettl14, Wtap, Alkbh5, Fto, and Ythdf1 expression, as shown by western blots of the glomeruli isolated from 5‐month‐old mice (Figure 2d; Figure S2e, Supporting Information). The resulting *Mettl3* ^podKO^ mice were born at the expected Mendelian frequency, without any gross renal anomalies in the newborns. At 2 months of age, no significant differences in proteinuria, serum creatinine, low‐density lipoprotein (LDL) levels, and histological features were observed between wild‐type (WT) and *Mettl3* ^podKO^ mice (Figure S2f–i, Supporting Information). However, transmission electron microscopy (TEM) revealed mild alterations in podocyte ultrastructure, characterized by foot process flattening and effacement in *Mettl3* ^podKO^ mice (Figure S2i, Supporting Information, bottom panels).

To investigate whether ablation of *Mettl3* in podocytes leads to more severe renal phenotypes, we compared renal function, proteinuria, and kidney histology in wild‐type (WT) and *Mettl3* ^podKO^ mice at 5 months of age. Podocyte *Mettl3* knockout led to marked increases in 24 h urine protein excretion, serum creatinine, and LDL levels (Figure 2e–g), accompanied by severe glomerular sclerosis, capsular adhesions, tubular dilation, and protein casts (Figure 2h, first to third panels). Based on the morphological features of FSGS, we identified various histological variants, including perihilar, cellular, and tip forms, in *Mettl3* ^podKO^ mice (Figure S3a, Supporting Information). Electron microscopy revealed that 5‐month‐old *Mettl3* ^podKO^ mice developed severe foot process effacement and mesangial cell proliferation (Figure 2h, fourth panel; Figure S3b,c, Supporting Information). In addition, the *Mettl3* ^podKO^ mice exhibited a significant increase in sclerotic glomeruli (Figure 2i), along with a marked reduction in glomerular size (Figure 2j) and RNA m6A methylation levels (Figure 2k; Figure S3d, Supporting Information). Consistent with increased glomerular sclerosis, the expression of slit diaphragm proteins, including nephrin and podocin, was reduced (Figure 2k,l; Figure S3e,f, Supporting Information). As expected, immunofluorescence examination of kidneys from the *Mettl3* ^podKO^ mice demonstrated that *Mettl3* expression was markedly decreased (Figure S4a,b, Supporting Information). In addition, glomerular expression of α‐SMA (Figure 2l; Figure S3g, Supporting Information) and fibronectin (Figure S4d,e, Supporting Information) was markedly increased. Together, these results demonstrate that the podocyte‐specific deletion of *Mettl3* leads to a marked reduction in glomerular RNA m6A methylation and recapitulates the typical phenotype of FSGS.

### 3.3 Deletion of Mettl3 Leads to Downregulation of the Podocyte Tight Junction Protein 1

To further decipher the mechanism by which podocyte deletion of *Mettl3* contributes to FSGS, RNA‐sequencing (RNA‐seq) and m6A‐immunoprecipitated RNA sequencing (MeRIP‐seq) were performed on purified podocytes from 5‐month‐old wild‐type and *Mettl3* ^podKO^ mice. RNA‐seq results showed that 3968 mRNAs were significantly dysregulated in podocytes (negative binomial regression analysis, p<0.05), including 2279 upregulated and 1689 downregulated transcripts (Figure 3a). KEGG bioinformatics analysis revealed that many significantly downregulated genes were enriched in the tight junction function pathway (46 genes; Figure S5a, Supporting Information). Importantly, significantly altered genes were also enriched in the tight junction pathway according to KEGG analysis (58 genes; Figure S5b, Supporting Information). These results suggest that podocyte tight junction assembly may be a primary target in the *Mettl3* ^podKO^ mice. Because mature podocytes lack tight junctions, which are replaced by the slit diaphragm (SD), we anticipated that deletion of *Mettl3* might cause podocyte SD dysfunction. Using key driver gene analysis (KDA) based on protein–protein interactions (PPI), we found that 40 of the significantly altered genes were KDA genes (Figure S5c, Supporting Information). KEGG analysis of these 40 KDA genes also highlighted tight junctions as a significantly enriched pathway (Figure S5d, Supporting Information). Furthermore, alignment of the KDA genes with the MeRIP‐seq data revealed six transcripts that overlapped and were significantly reduced in the *Mettl3*‐deficient podocytes (Figure 3b,c). The PPI interaction analysis and KEGG pathway relationship network analysis of the six genes also identified tight junctions as a significantly enriched pathway, with tight junction protein 1 (*Tjp1*; 7 gene node connections) and claudin 1/16 (*Cldn1/16*; 5 gene node connections) being mostly involved (Figure S5e, Supporting Information; Figure 3d). We further verified the decreased protein expression of Tjp1 and increased Cldn1/16 protein levels in the glomeruli isolated from WT and *Mettl3* ^podKO^ mice (Figure 3e). In line with the western blot results, histological detection also confirmed the reduced Tjp1 and increased Cldn1 expression in the *Mettl3* ^podKO^ mice compared with WT mice (Figure 3f; Figure S5f,g, Supporting Information). Since Cldn1 and 16 are normally expressed in the parietal epithelial cells of Bowman's capsule and the thick ascending limb of the loop of Henle, respectively,^[^ ^27^, ^28^ ^]^ we speculate that dysregulation of Cldn1/16 may be a secondary effect in *Mettl3* deficient podocytes, and hence, it reminds us that TJP1 may be the main target of *Mettl3* in podocytes. In support of this, a luciferase assay using 293A cells showed that *Mettl3* could bind to *TJP1* mRNA (Figure 3h). Furthermore, based on the amino acid sequence and structural analysis of Mettl3 (http://abragam.med.utoronto.ca/cgi‐bin/cal_seq_all.py;www.uniprot.org) (Figure S6a,b, Supporting Information), the C‐terminal F3 fragment between amino acid 357–580 in the Mettl3 protein was found to be the main binding site for Tjp1 (Figure S6c, Supporting Information). Moreover, an RNA immunoprecipitation assay (RIP) was used to pull down RNA in cultured podocytes using an antibody against GFP‐tagged Mettl3, and a qPCR analysis for *T*JP*1*, *Tmem30*, and *Cldn1*. The results showed that *Mettl3* increased the mRNA levels of *Tjp1* but not those of *Tmem30* and *Cldn1* (Figure 3i). Using HCR analysis, we further showed that *Mettl3* could directly bind to *Tjp1* mRNA (Figure 3J). To further confirm the effect of *Mettl3* on Tjp1 in podocytes, we treated podocytes with Mettl3 siRNA for 24h and 48h and determined cell viability and *Tjp1* mRNA level. As determined by the CCK‐8 assay, podocyte cell viability was impaired after *Mettl3* gene silencing (Figure 3k), with a significantly shortened half‐life of *Tjp1* mRNA (Figure 3l). These results suggest that *Mettl3*‐mediated RNA m6A modification of *Tjp1* promotes mRNA stability. Collectively, these findings demonstrate that *Tjp1* is a potential target of *Mettl3* in podocytes, where *Mettl3* increases *Tjp1* m6A modification by stabilizing its expression.

### 3.4 Mettl3 Induces CDC42 Expression Through TJP1 in Podocytes

**Figure 4:**
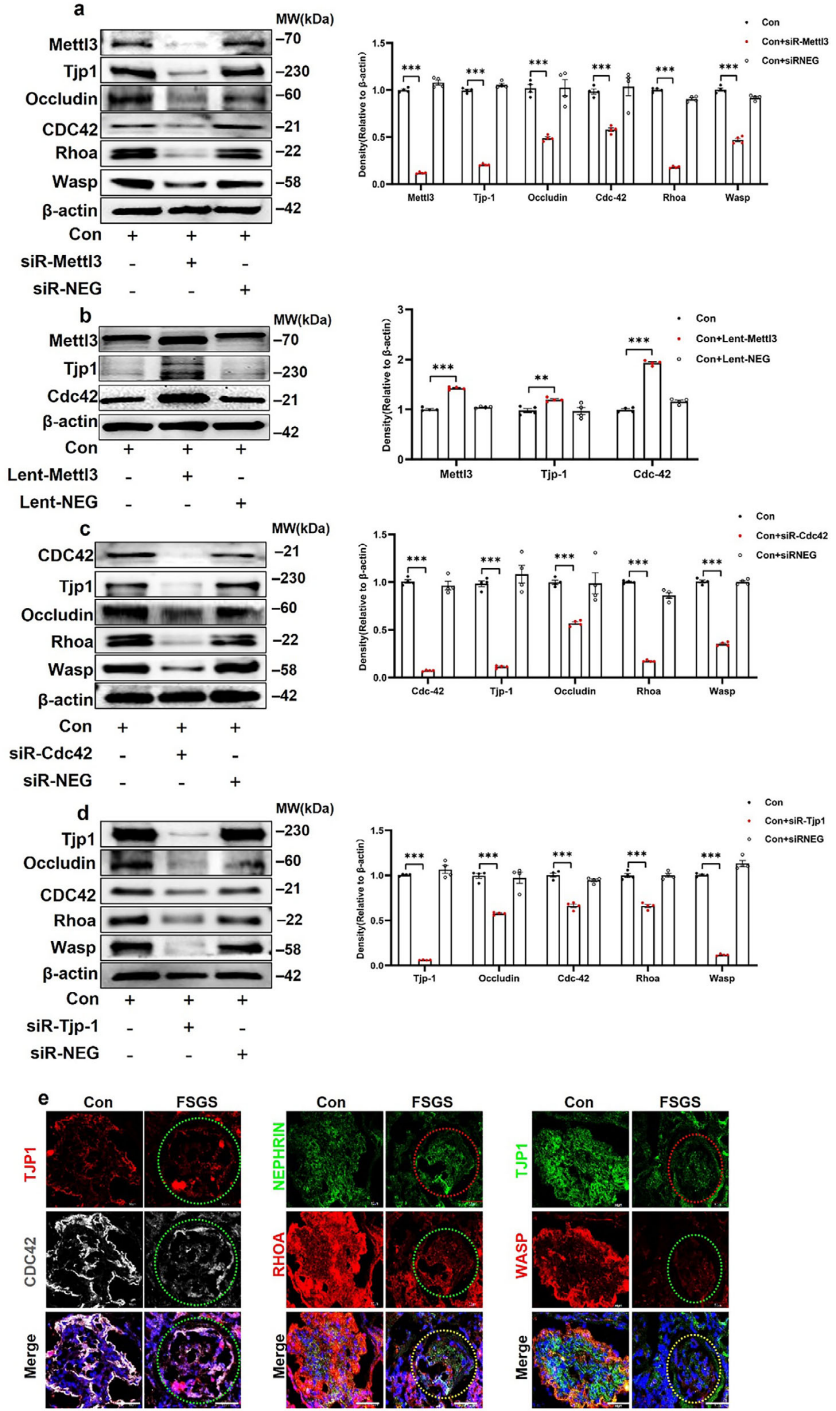
Mettl3 binds to Tjp1 to regulate *CDC42*. a,b) Representative western blot analyses of Mettl3, Tjp1, Occludin, Cdc42, and Rhoa or Wasp in MPC cells transfected with *Mettl3* lentivirus and siRNA, respectively (n = 4). The right panels show quantification of the expression levels. c,d) Representative western blot analysis of Tjp1, Occludin, Cdc42, and Rhoa or Wasp in MPC cells transfected with *Cdc42* or *Tjp1* siRNA (n = 4). The right panels show quantification of the expression levels. e) Representative immunofluorescent images of TJP1 and CDC42/RHOA/WASP kidney tissue sections from patients with FSGS (n = 14, Scale bar = 50 µm). ^*^ *p* < 0.05 and ^***^ *p* < 0.001. Data are shown as mean ± SD.

To further characterize the role of the Mettl3/Tjp1 axis in the podocytes of the *Mettl3* ^podKO^ mice, we used a bioinformatics approach to analyze the relationship between the six genes in the tight junction cluster (Figure 3b,c). We found that Cldn1 and Tjp1 were more interactive in the subcluster of “Claudin and Tricellulin” of the tight junction KEGG pathway (Figure S7a, Supporting Information). Among these, the *Cdc42* gene was found to be most closely related to *Tjp1*. Simultaneously, we showed that the expression of CDC42 and its related genes, including Rhoa and Wasp, decreased in ADR‐treated MPC cells (Figure S7b, Supporting Information). As it has been previously reported that Cdc42 is involved in the pathological process of impaired podocytes,^[^ ^29^ ^]^ we tested the effect of Mettl3 on Cdc42 in podocytes. Primary cultured podocytes were infected with *Mettl3* lentivirus or transfected with *Mettl3* siRNA (**Figure** **4**a,b). Knockdown of *Mettl3* resulted in a marked reduction in both Tjp*1* and Cdc42 levels (Figure 4a). Similarly, the expression of Tjp1 and Cdc42 downstream genes, including occludin, Rhoa, and Wasp, significantly decreased (Figure 4a). Conversely, *Mettl3* overexpression increased Tjp1 and Cdc42 expression (Figure 4b). Furthermore, knockdown of *Cdc42* resulted in reduced Tjp1 expression (Figure 4c), and silencing *Tjp1* led to a significant decrease in Cdc42 expression (Figure 4d). Consistent with previous findings, these results demonstrate that *Mettl3* overexpression increases, while *Mettl3* knockdown decreases Tjp1 expression in podocytes. In support of this, immunofluorescence study demonstrated that, along with reduced Tjp1 expression, the expression of Cdc42 and its related genes Rhoa and Wasp was markedly decreased in renal glomeruli of patients with FSGS (Figure 4e; Figure S7c–g, Supporting Information).

### 3.5 Administration of N6‐Methyladenosine Ameliorates FSGS in the Mettl3^podKO^ Mice

**Figure 5:**
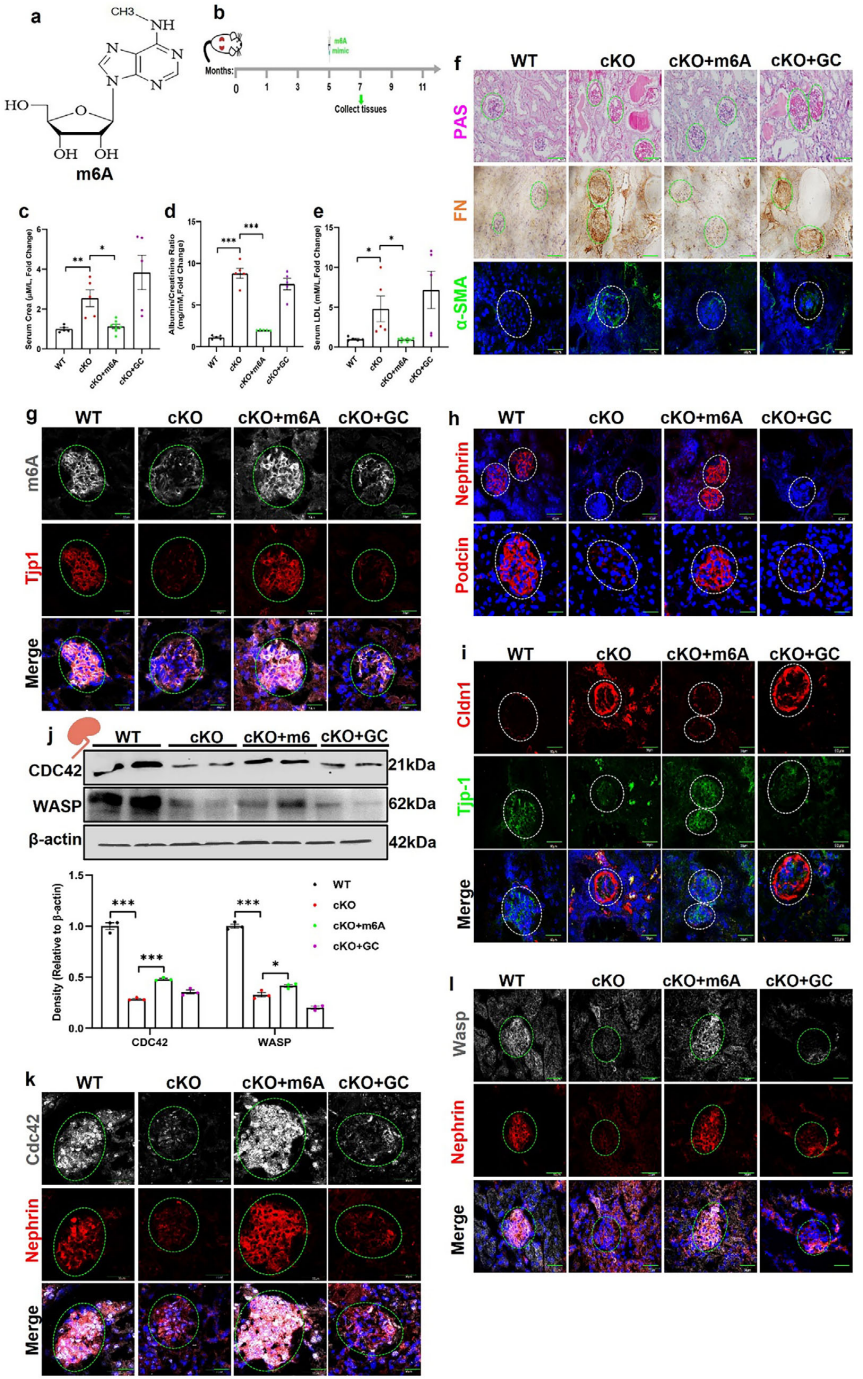
Administration of N6‐methyladenine ameliorates the progression of FSGS in the *Mettl3* ^podKO^ mice. a) Molecular structures of m6A mimic chemicals. b) Protocol for the m6A mimic chemical treatment of *Mettl3^podKO^* mice. c–e) Quantification of serum creatinine and LDL or proteinuria in m6A chemical‐and glucocorticoid (GC) treated *Mettl3^podKO^* mice compared to non‐treated *Mettl3^podKO^* mice (n = 5). f) Representative images of PAS, FN, and α‐SMA staining in kidney sections of m6A and GC‐treated *Mettl3^podKO^* mice (n = 5, Scale bar = 40 µm). g) Representative immunofluorescence images of m6A and Tjp1 in kidney sections from m6A‐ and GC‐treated *Mettl3^podKO^* mice (n = 5, Scale bar = 40 µm). h) Representative immunofluorescent images of nephrin and podocin in kidney sections from m6A‐ and GC‐treated *Mettl3^podKO^* mice (n = 5, Scale bar = 40 µm). i) Representative immunofluorescence images of Tjp1 and Cldn1 in kidney sections from m6A‐ and GC‐treated *Mettl3^podKO^* mice (n = 5, Scale bar = 40 µm). j) Representative western blot analysis of Cdc42 and Wasp in m6A chemical and GC‐treated *Mettl3^podKO^* mice compared to WT and non‐treated *Mettl3^podKO^* mice (n = 3, lower panel shows quantification). k,l) Representative immunofluorescent images of Cdc42, Wasp, and nephrin in kidney sections from m6A‐ and GC‐treated *Mettl3^podKO^* mice (n = 5,Scale bar = 40 µm). ^*^ *p* < 0.05; ** *p* < 0.01 and *** *p* < 0.001. Data are shown as mean ± SD.

As shown above, deletion of *Mettl3* reduced RNA m6A modifications in podocytes, leading to FSGS in the *Mettl3* ^podKO^ mice. Next, we tested whether exogenous administration of N6‐methyladenosine, an RNA m6A mimic (**Figure** **5**a), could rescue the FSGS phenotype in the *Mettl3* ^podKO^ mice. We treated 5‐month‐old *Mettl3* ^podKO^ mice with N6‐methyladenosine for two months (Figure 5b). Treatment with N6‐methyladenosine significantly improved kidney function (Figure 5c), and attenuated proteinuria (Figure 5d) and serum LDL levels (Figure 5e) in the *Mettl3* ^podKO^ mice. Administration of N6‐methyladenosine markedly ameliorated the glomerular sclerosis, tubular casts, fibronectin (FN) accumulation, and α‐SMA expression (Figure 5f; Figure S8a,b, Supporting Information). As expected, N6‐methyladenosine treatment restored glomerular RNA m6A levels and the glomerular expression of Tjp1, nephrin, and podocin (Figure 5g,h; Figure S8c–f, Supporting Information). In contrast, the expression of Cldn1 was reduced in the *Mettl3* ^podKO^ mice receiving N6‐methyladenosine (Figure 5i; Figure S8g, Supporting Information). Furthermore, we measured Cdc42 and Wasp protein expression using western blotting and found a significant increase in both Cdc42 and Wasp expression after N6‐methyladenosine treatment in the *Mettl3* ^podKO^ mice (Figure 5j). Consistently, the increased expression of Cdc42 and Wasp was also evident after N6‐methyladenosine treatment in *Mettl3* ^podKO^ mice, as assessed by immunofluorescence measurements (Figure 5k,l; Figure S8h,i, Supporting Information). Together, these findings demonstrate that administration of N6‐methyladenosine ameliorates FSGS via restoring glomerular RNA m6A levels and the glomerular expression of Tjp1, nephrin, and podocin in the *Mettl3* ^podKO^ mice.

### 3.6 Treatment with N6‐Methyladenosine Attenuated Adriamycin‐Induced FSGS

**Figure 6:**
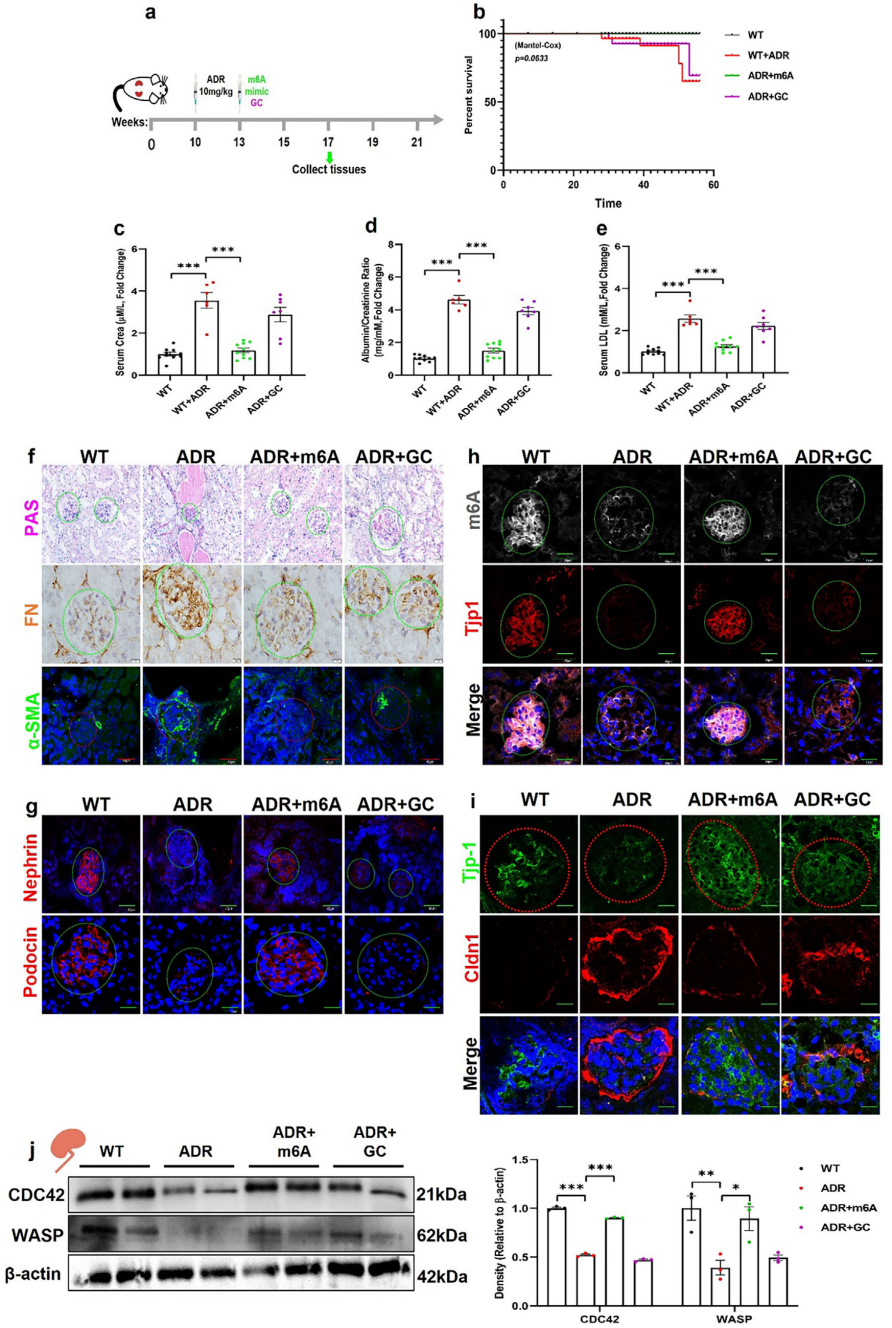
Administration of N6‐methyladeine ameliorates the progression of FSGS in ADR‐induced FSGS mice. a) Protocol for the m6A chemical treatment of ADR‐injected mice (n = 10). b) Survival curves of m6A (n = 10) and GC‐treated ADR‐induced FSGS mice (n = 7), showing that m6A significantly improved the survival rate of ADR‐induced mice. c–e) Quantification of serum creatinine, LDL, and proteinuria in m6A (n = 10) and GC‐treated ADR‐induced FSGS mice (n = 7) compared to untreated FSGS mice (n = 6). f) Histological analysis of PAS, FN, and α‐SMA staining in kidney sections of m6A and GC‐treated ADR‐induced mice compared to WT and non‐treated FSGS mice (n = 6, Scale bar = 40 µm). g) Representative immunofluorescence images of nephrin (Scale bar = 40 µm) and podocin (Scale bar = 20 µm) in kidney sections of m6A‐ and GC‐treated FSGS mice compared with those of WT and non‐treated FSGS mice (n = 6). h) Representative immunofluorescence images of RNA m6A levels and Tjp1 expression in kidney sections of m6A‐ and GC‐treated FSGS mice compared to WT and untreated FSGS mice (n = 6, Scale bar = 40 µm). i) Representative images of Tjp1 and Cldn1 immunofluorescent staining in kidney sections of m6A‐ and GC‐treated FSGS mice compared to those of WT and untreated FSGS mice (n = 6, Scale bar = 20 µm). j) Representative western blot for Cdc42 and Wasp in m6A‐ and GC‐treated ADR‐induced FSGS mice compared to WT and untreated FSGS mice (n = 3; right panel shows quantification). ^*^ *p* < 0.05; ^**^ *p* < 0.01 and ^***^ *p* < 0.001. Data are shown as mean ± SD.

To further confirm the beneficial effect of N6‐methyladenosine on FSGS, we generated a mouse model of FSGS using Adriamycin (**Figure** **6**a). We found that 4‐week N6‐methyladenosine treatment significantly increased the survival rate of mice treated with ADR (Figure 6b). N6‐methyladenosine treatment also significantly lowered serum creatinine levels, urine protein excretion, and serum LDL levels (Figure 6c–e). PAS staining, immunohistochemistry, and immunofluorescence studies further showed that glomerular sclerosis, tubular injury, and expression levels of FN and α‐SMA were markedly attenuated in N6‐methyladenosine‐treated ADR mice (Figure 6f; Figure S9a,b, Supporting Information). Similarly, glomerular RNA m6A levels, Tjp1, podocin, and nephrin expression levels were restored after N6‐methyladenosine treatment (Figure 6g,h; Figures S9c–f, S10b, Supporting Information). In addition, ADR‐promoted Cldn1 expression was attenuated by N6‐methyladenosine treatment (Figure 6i; Figure S9g, Supporting Information). The levels of m6A related enzymes Mettl3 and Mettl14, Wtap, Alkbh5, and Ythdf1 increased after N6‐methyladenosine treatment (Figure S10a,c, Supporting Information). Furthermore, N6‐methyladenosine treatment significantly upregulated Cdc42 and Wasp expression in the ADR‐treated mice (Figure 6j). Collectively, these results demonstrate that the exogenous administration of N6‐methyladenosine can improve renal function and attenuate albuminuria and renal fibrosis in ADR‐induced FSGS.

## 4 Discussion

In the present study, we demonstrated that levels of N6‐methyladenosine are reduced in renal glomeruli of patients and mice with FSGS. Podocyte‐specific deletion of *Mettl3* results in a significant reduction in glomerular RNA m6A levels and a typical FSGS phenotype in mice. RNA‐seq studies show that *Mettl3* knockdown markedly affects the tight junction‐related pathways in podocytes, where Mettl3 binds to Tjp1 mRNA to increase its stability. Moreover, N6‐methyladenosine treatment not only ameliorates the pathogenesis of FSGS in the *Mettl3* ^podKO^ mice but also improves ADR‐induced FSGS. Collectively, Mettl3 is essential for podocyte function and represents an attractive target for FSGS treatment.

During kidney development, mature podocytes undergo terminal differentiation to form slit diaphragms (SDs) between the opposing foot processes. The dysfunction of the actin cytoskeleton in podocytes disrupts the architecture of the glomerular basement membrane (GBM), leading to impaired filtration properties and proteinuria. In this study, we demonstrated that Mettl3 binds directly to Tjp1 and markedly increases Tjp1 expression, possibly by enhancing Tjp1 mRNA stability. It is well known that Tjp1 is a component of the slit diaphragm, thereby regulating the podocyte filtration barrier. As previously reported, the podocyte‐specific deficiency of *Tjp1* downregulates the expression of podocyte membrane proteins and impairs the interdigitation of foot processes and formation of the slit diaphragm, resulting in global glomerulosclerosis.^[^ ^9^ ^]^ Therefore, our findings demonstrate that *Mettl3* deficiency in podocytes leads to FSGS, possibly by suppressing *TJP1* expression.

To further clarify the molecular mechanisms underlying Tjp1 defect‐associated podocyte impairment, we utilized bioinformatics and molecular biology approaches to investigate the downstream biological processes of Tjp1. We found that Tjp1 plays an important role in regulating the levels of Cdc42, a member of the Rho family of small guanosine triphosphatases (GTPases), which is critical for the polymerization of actin at sites of nephrin aggregates in podocytes.^[^ ^29^ ^]^ Silencing Tjp1 resulted in a striking reduction in Cdc42 levels in podocytes, suggesting an important role for Tjp1 in the regulation of actin cytoskeleton plasticity. Simultaneously, it has been reported that Tjp1 directly binds to MRCKb, a Cdc42 effector kinase that modulates cell protrusion and migration, at the leading edge of migrating cells.^[^ ^30^ ^]^ Previous studies have reported that podocyte‐specific loss of Cdc42 leads to congenital nephropathy with massive proteinuria due to the extensive effacement of podocyte foot processes with abnormal junctional complexes,^[^ ^29^ ^]^ suggesting that Cdc42 plays a crucial role in regulating the dynamics of the actin cytoskeleton in podocytes and maintaining the podocyte filtration barrier.^[^ ^29^ ^]^ The Wiskott–Aldrich syndrome protein (WASP/N‐WASP) belongs to the family of nucleation‐promoting factors. It is considered a downstream effector of Cdc42^[^ ^31^ ^]^ and is implicated in the stabilization of podocyte foot processes.^[^ ^32^ ^]^ Indeed, in the present study, we found that the levels of Wasp in the renal glomeruli were markedly reduced in both *Mettl3* ^podKO^ and ADR‐treated mice, which could be reversed by treating the mice with m6A mimics. Together, these findings demonstrate that dysregulated Tjp1 expression may mediate *Mettl3* deficiency‐induced FSGS by inhibiting the function of the Cdc42/Wasp axis in podocytes.

In the present study, we found that glomerular RNA m6A modifications were markedly repressed in both patients and mice with FSGS. We also found that podocyte‐specific deletion of *Mettl3* resulted in a significant decrease in glomerular RNA m6A modifications. These observations suggested that RNA m6A methylation plays a critical role in the pathogenesis of FSGS. In support of this, treatment of both *Mettl3* ^podKO^ and ADR‐treated mice with m6A mimics markedly increased the levels of glomerular RNA m6A modifications and significantly attenuated proteinuria and glomerular sclerosis. These findings provide direct evidence that exogenous supplementation with m6A is an attractive strategy for the treatment of FSGS.

We noted that, in contrast to the findings in the present study, it has also been reported that elevated Mettl3 or Mettl14‐mediated m6A RNA levels are associated with the pathogenesis of various kidney diseases. Mettl3 expression is increased in mice with DN.^[^ ^19^ ^]^ Mettl3‐induced RNA m6A modification was also found to be increased in autosomal dominant polycystic kidney disease (ADPKD), and deletion of Mettl3 in renal tubular epithelial cells improved ADPKD in mice.^[^ ^18^ ^]^ Additionally, Mettl3 is implicated in acute kidney injury (AKI), and inhibition of Mettl3 is able to alleviate the advancement of acute kidney injury.^[^ ^33^ ^]^ Recently, another report demonstrated elevated Mettl14 and m6A levels in FSGS and DN.^[^ ^20^ ^]^ These findings show that the function of Mettl3 was mainly involved in renal tubules, and they suggest that targeting Mettl3 or Mettl14‐mediated mRNA post‐transcriptional modifications may represent a promising approach for the treatment of AKI, FSGS, DN, and ADPKD. In the present study, our study also showed that Mettl3 and its downstream molecules, such as Tjp1 and Cdc42, were not only expressed in podocytes but in renal tubules and interstitium (Figure 5k; Figure S10a, Supporting Information), deletion of Mettl3 had no obvious kidney disease phenotypes before 2 months of age, however it exhibited a severe FSGS‐like phenotype at 5 months of age, which may reflect very different stages of pathogenesis with different expression levels of m6A and Mettl3/14. This was consistent with our previous report on the role of miR‐25 in heart failure.^[^ ^34^ ^]^ Therefore, the role of Mettl3 may vary depending on the cell type, stage, and type of kidney disease. Further studies are needed to understand the precise role of Mettl3 and other RNA m6A methylation‐catalyzing enzymes in regulating podocyte maturation and function, and in the pathogenesis of various renal disorders.

In summary, the present study reports a critical role for Mettl3‐mediated RNA m6A modification in the maintenance of podocyte architecture and the pathogenesis of FSGS. Podocyte‐specific Mettl3 deletion results in reduced levels of glomerular RNA m6A modification and a typical FSGS phenotype. Exogenous supplementation with an m6A mimic restores glomerular RNA m6A methylation levels and attenuates glomerulosclerosis and proteinuria in animal models of FSGS. Mettl3 appears to be a potential therapeutic target for the treatment of FSGS.

## Conflict of Interest

The authors declare no conflict of interest.

## Supporting information



Supporting Information

Supporting Information

## Data Availability

The data that support the findings of this study are openly available in deficient of mettl3‐induced m6A causes podocyte impairment in mice at https://www.ncbi.nlm.nih.gov/search/all/?term=PRJNA1017102, reference number 1017102.
