# Supplementary material for: Mettl3‐Mediated m6A Modification Represents a Novel Therapeutic Target for FSGS
Source: Adv Sci (Weinh). 2025 Dec 19;13(10):e01242. doi: 10.1002/advs.202501242 (PMC12915192; doi:10.1002/advs.202501242)
Supplement: Supplementary file 1 — Supporting Information [file ADVS-13-e01242-s001.docx]

**Supplementary Figure Legands**

**Figure.S1 Mice treated with adrimycin exhibit a FSGS-like phenotype**

**a**,**b)** Quantification of proteinuria and serum creatinine levels in WT and adrimycin (ADR)-treated mice (n = 6). **c**) Representative images of HE and PAS staining of kidney sections from WT and ADR-treated mice (n = 6, Scale bar = 40μm). **d**-**i**) Quantification of immunofluorescence analysis for m6A, Mettl3/14, Wtap, podocin and Tjp1 levels were derived based on main Figure1a-d; **j**) Quantification of cell viability by the CCK8 assay in MPC cells treated with ADR for 12–72 h (n = 6). **k**) Representative western blot analysis of Wtap, Mettl3 and 14, WT-1, SYNPO and Nephrin in the MPC cells treated with ADR for 12–72 h (n = 4). **l**-**q**) Quantification of immunofluorescence analysis for m6A, Mettl3/14, Wtap, nephrin, and Tjp1 levels were performed based on main Figure 1j–l. * *p* < 0.05, ** *p* < 0.01 and *** *p* < 0.001. Data are shown as mean ± SD.

**Figure.S2 Deletion of *Mettl3* in the podocyte results in flattening of podocyte foot processes at 2-Month-old mice**

**a)** Genotyping of the *Mettl3*^flox/flox^, NPHS2-Cre, and *Mettl3*^podKO^ mice. **b)** Quantification of *Mettl3* mRNA expression in cultured podocytes from glomeruli isolated from WT and *Mettl3*podKO mice (n = 6). **c**, **d**) Immunofluorescence levels of Mettl3 and Tjp1 were quantified based on main Figure 2c. **e**) Quantification of the western blot analysis for Mettl14, Wtap, Alkbh5, Fto, and Ythdf1 levels were obtained based on main figure 2d (n = 4). **f**–**h)** Quantification of proteinuria, serum creatinine, and LDL in 2-month-old WT and *Mettl3*^podKO^ mice (n = 7). **i**) Representative images of HE (Scale bar = 40μm), PAS (Scale bar = 40μm), and TEM (Scale bar = 2μm) staining of kidney sections from WT and *Mettl3*^podKO^ mice at 2-month of age (n = 6). ** *p* < 0.01 and *** *p* < 0.001. Data are shown as mean ± SD

**Figure.S3 Deletion of *Mettl3* in the podocyte results in FSGS at 5-Month-old mice**

**a)** Representative images of PAS staining of 5-month-old *Mettl3*^podKO^ mice showing deficiency of *Mettl3* in podocytes, resulting in different subclassifications of FSGS, including perihilar (green arrow: capsular synechia, black arrow: segmental sclerosis), cellular (green arrow: foam cells, yellow arrow: capsular synechia, black arrow: segmental sclerosis) , and tip variants (green arrow: scapillary loops and proximal tubule synechia and black arrow: segmental sclerosis , n = 6, Scale bar = 40μm). **b**) Representative images of the TEM assay from 5-month-old *Mettl3*^podKO^ mice (n = 6, scale bar = 10μm), which showed that knocking out the *Mettl3* gene in podocytes led to a narrowed capillary lumen, mesangial cell proliferation, podocyte foot process effacement, and complete loss of slit diaphragms in *Mettl3*^podKO^ mice compared to WT mice. Podocytes (p), capillary lumen (c), erythrocytes (e), and mesangial cells (m).**c**) Quantification of mesangium cells number based on Figure.S3b. **d**-**g**) Quantification of immunofluorescence analysis for m6A, podocin, nephrin and α-SMA levels were obtained based on main Figure 2k and l. ** *p* < 0.01 and *** *p* < 0.001. Data are shown as mean ± SD.

**Figure.S4 Deletion of podocyte *Mettl3* results in downregulation of *Mettl3* and *TJP1* expression in mice at the age of 5 months.**

**a-c)** Representative images of Mettl3 and Tjp1 expression in the glomeruli of 5-month-old *Mettl3*^podKO^ and WT mice (n = 6, Scale bar = 40μm), b and c) panels show quantification of Mettl3 and TJP1 levels. **d**) Immunohistochemical analysis of fibronectin (FN) expression in the glomeruli of 5-month-old WT and *Mettl3*^podKO^ mice (n = 6, Scale bar = 40μm). **e**) panel shows quantification of FN levels. *** *p* < 0.001. Data are shown as mean ± SD.

**Figure.S5 Bioinformatics analysis of RNA-seq data in isolated podocytes from the WT and *Mettl3*^podKO^ mice**

**a**, **b)** KEGG bioinformatics analysis of significantly decreased and remarkably altered genes identified by RNA-seq in *Mettl3*^podKO^ mice compared to WT mice, respectively. **c**) Key driver gene analysis (KDA) showing 40 KDA genes in the podocytes of *Mettl3*^podKO^ mice compared to WT mice. **d**) KEGG analysis of 40 KDA genes demonstrating that these dysregulated genes were enriched in the tight junction term. **e**) Protein-protein interaction (PPI) network bioinformatics analysis of the six overlapping genes identified from the Venn diagram. **f**, **g)** Levels of TJP1 and Cldn1 were quantified based on main Figure 3 f and g. *** *p* < 0.001. Data are shown as mean ± SD.

**Figure.S6 the end sequence of *Mettl3* bind *TJP1***

**a**) *METTL3* protein amino acid sequence analysis. **b)** Molecular structure of the *Mettl3* protein. **c**: Luciferase activity in HEK 293A cells transfected with various fragmented *Mettl3* sequences and *TJP1* gene construct reporter confirmed that the end sequence of *Mettl3* (F3) is the main region for *TJP1* regulation (n = 4).

**Figure.S7 KEGG pathway map for tight junction**

**a)** CDC-42 is involved in tight junction KEGG terms. **b**) Representative western blot analysis of *CDC42*, Rhoa and Wasp or Occludin in the MPC cells treated with ADR, the under panel shows quantification (n = 4). **c**-**g**) Quantification of immunofluorescence analysis for Tjp1, Cdc-42, nephrin and Rhoa or Wasp levels were obtained from main Figure 4e. ** p < 0.01; *** p < 0.001. Data are shown as mean ± SD.

**Figure.S8 Administration of N6-methyladenosine ameliorates FSGS in the Mettl3podKO mice**

**a**-i**)** Expression levels of FN,α-SMA, m6A, Tjp1, nephrin and podocin, Cldn1, Cdc42 or Wasp were quantified based on main Figure. 5f, g, h, I, k, l, respectively. * p < 0.05; *** p < 0.001. Data are shown as mean ± SD.

**Figure.S9** **N6-methyladenine therapy attenuates the progression of ADR-induced FSGS phenotype.**

**a**-**g)** Quantification of FN, α-SMA, nephrin, podocin, m6A and Tjp1 or Cldn1 levels were performed derived from main Figure. 6f, g, h, i, respectively. *** p < 0.001. Data are shown as mean ± SD.

**Figure.S10 Treatment with N6-methyladenine ameliorates the progression of ADR-induced FSGS phenotype.**

**a**) Representative immunofluorescence images of Mettl3 and Tjp1 in the glomeruli of ADR-induced FSGS mice after treatment with m6A for 4weeks compared to untreated FSGS mice or GC-treated FSGS mice. **b**) Quantification of RNA m6A methylation levels in tissues, including the heart, liver, kidney, spleen, lung, brain, and testis from WT and m6A chemically treated ADR-induced FSGS mice compared to un-treated ADR-induced mice (n = 5). **c**) Representative western blot analysis of Wtap, Mettl3/14, Alkbh5, and Ythdf1 in tissues including the kidney and testis from WT and m6A chemicals treated ADR-induced FSGS mice compared to un-treated ADR-induced mice (n = 4). ** p < 0.01. Data are shown as mean ± SD.

**Supplemental Figure**


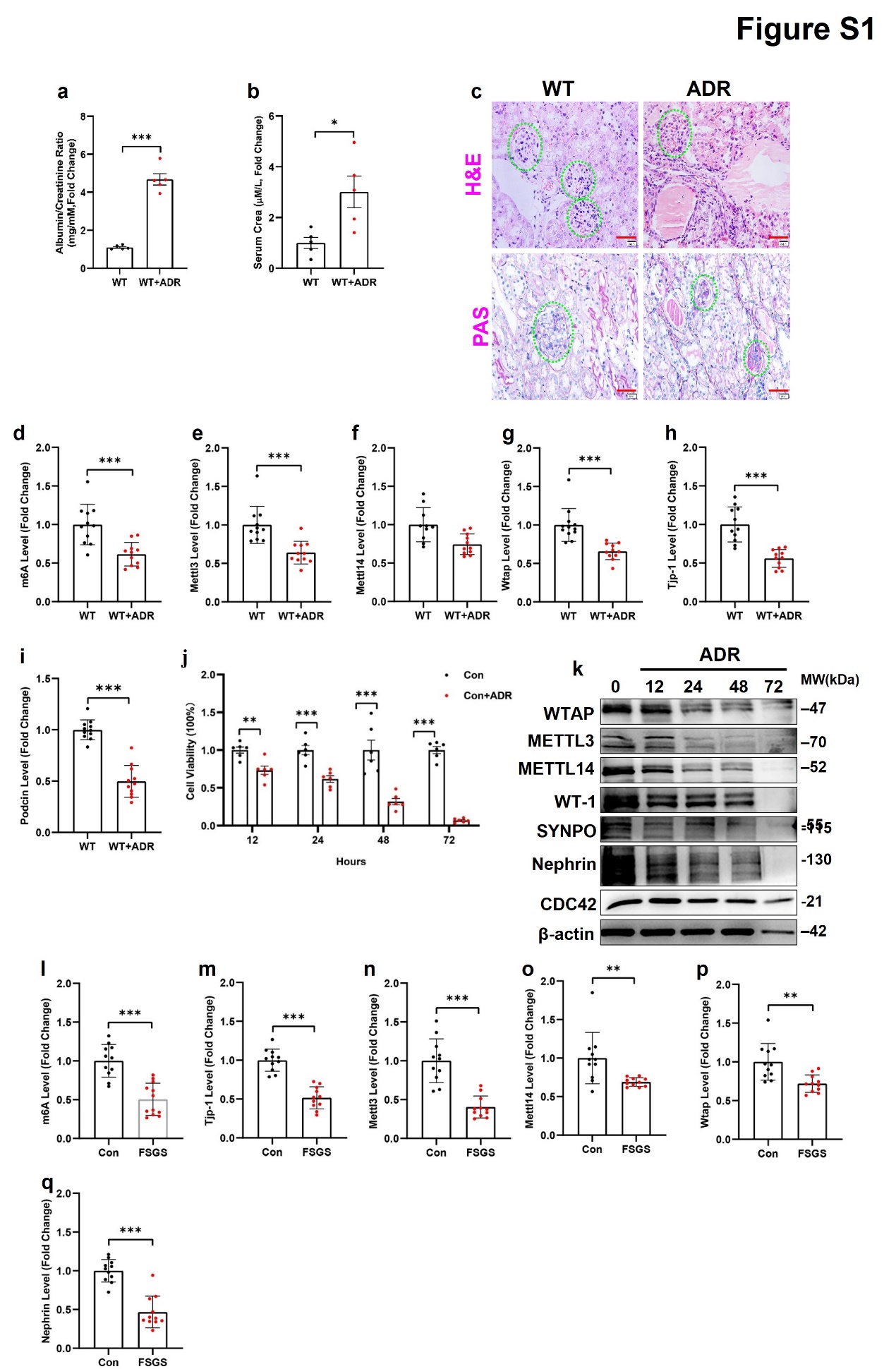


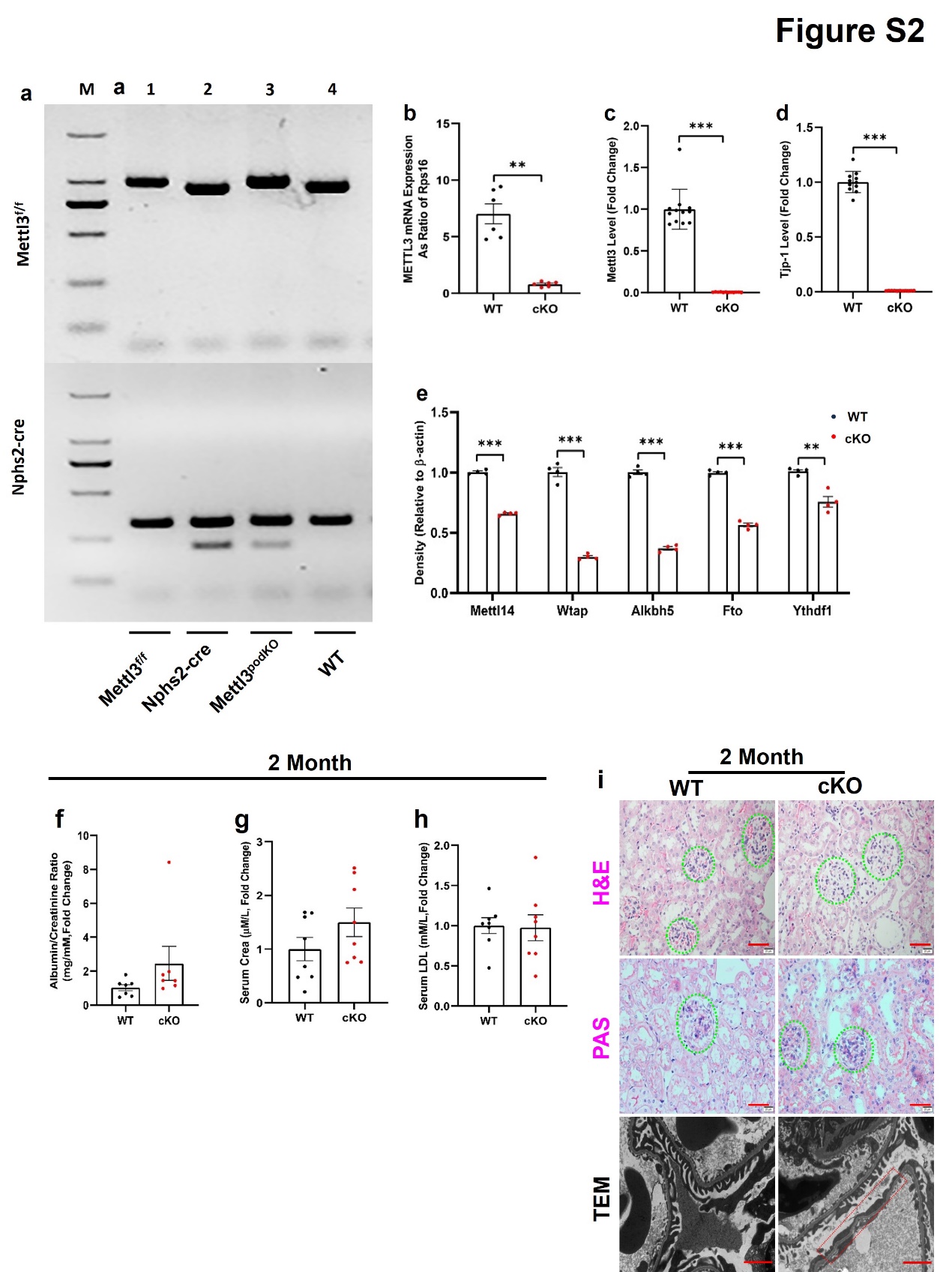


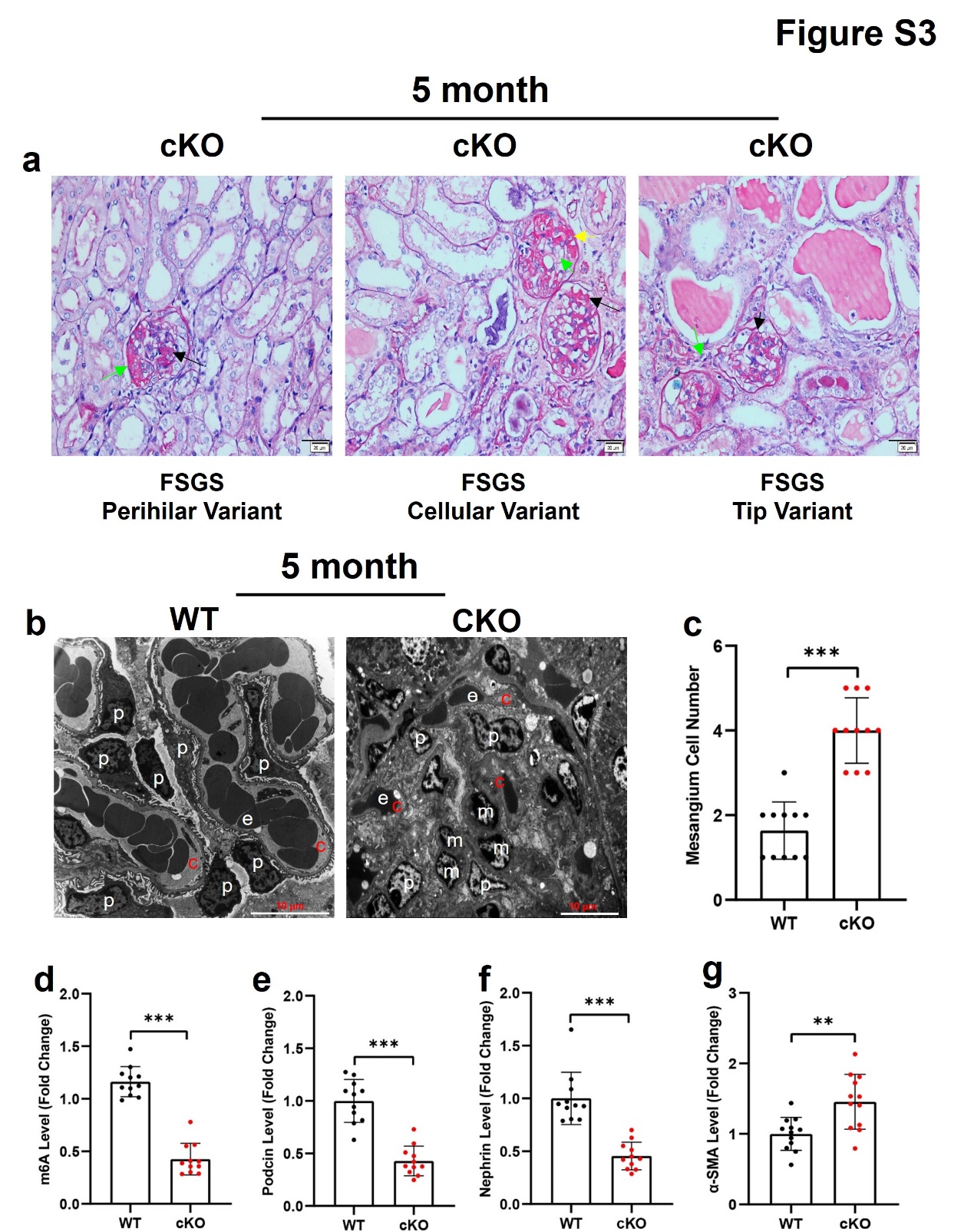


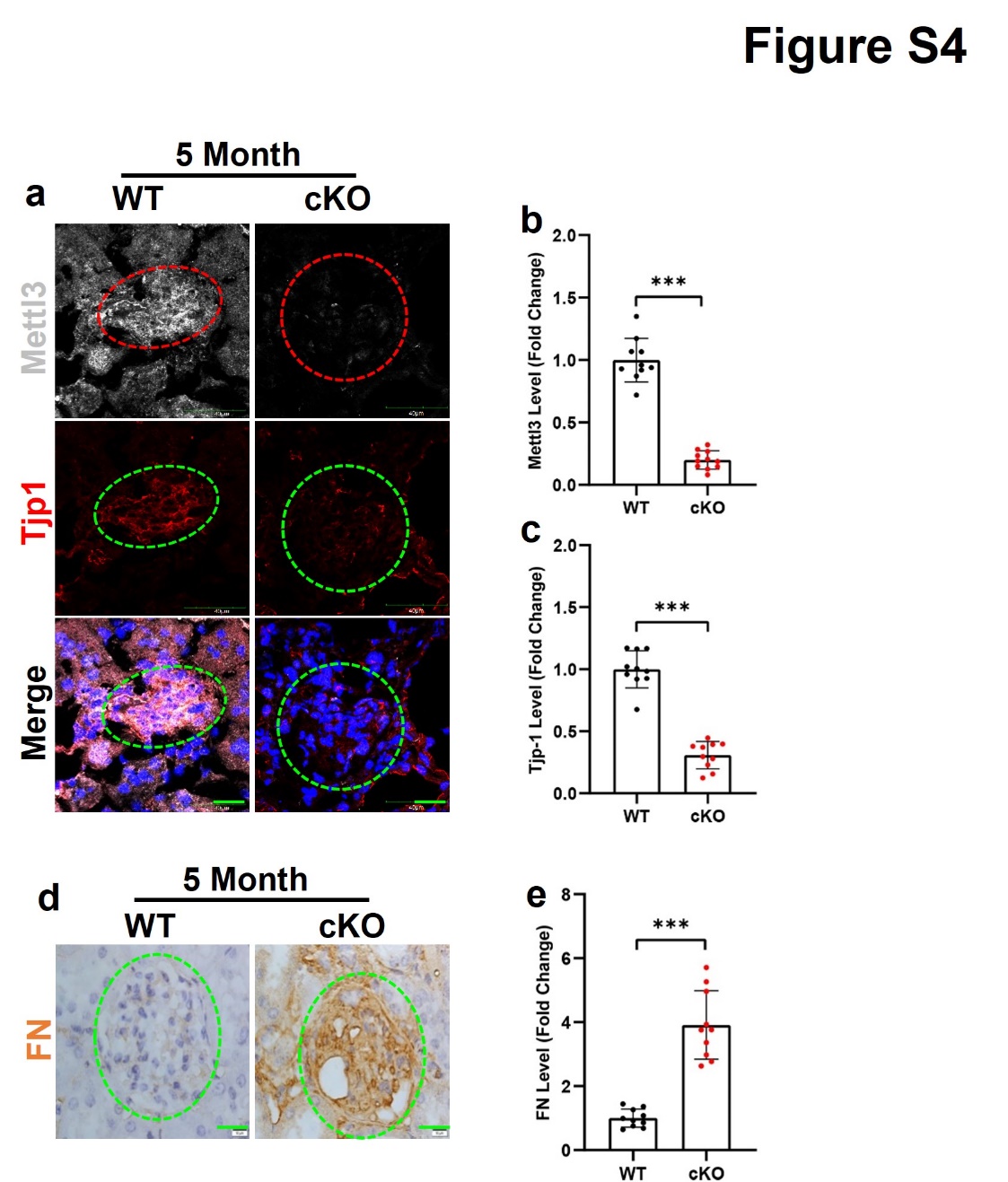


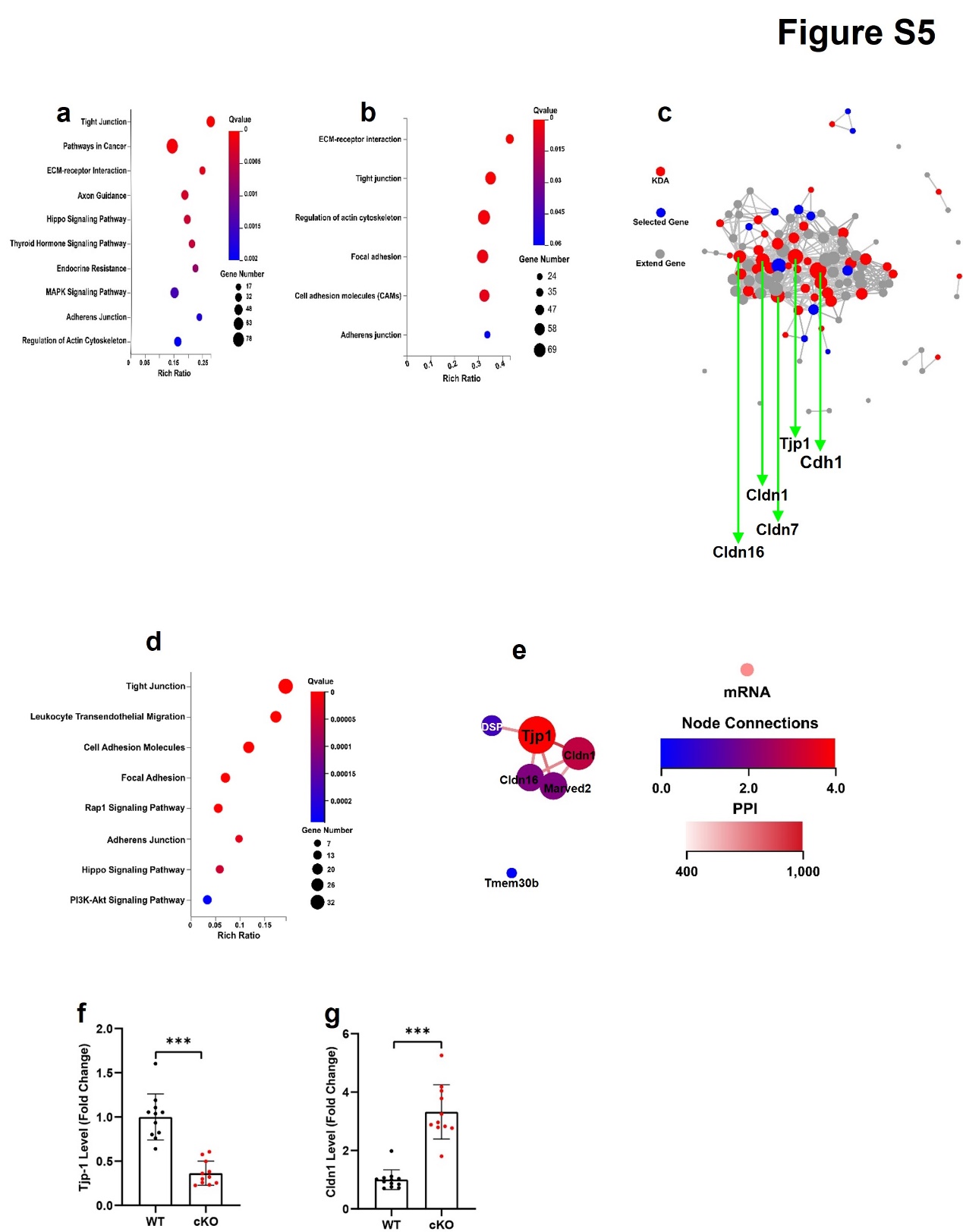


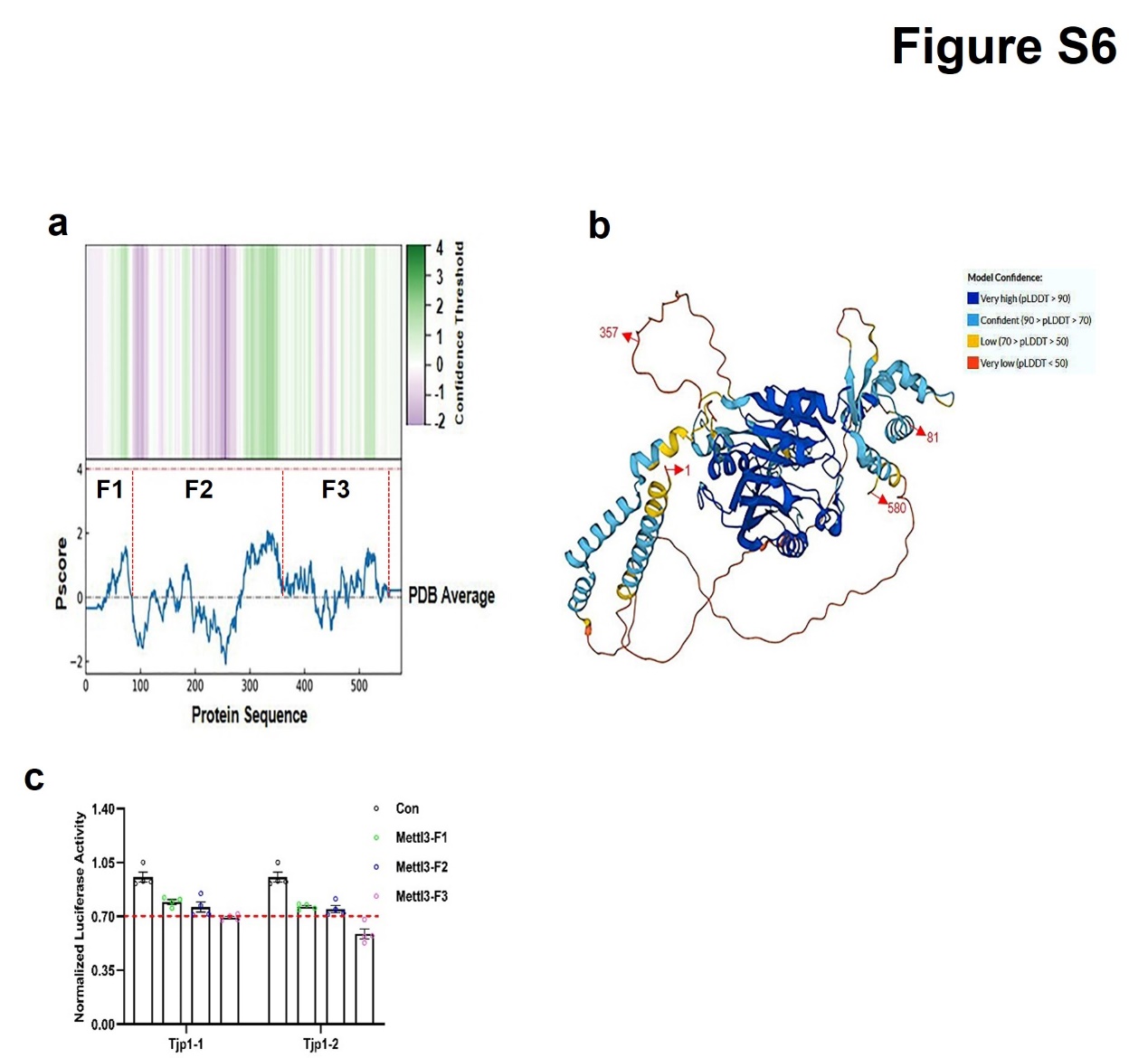


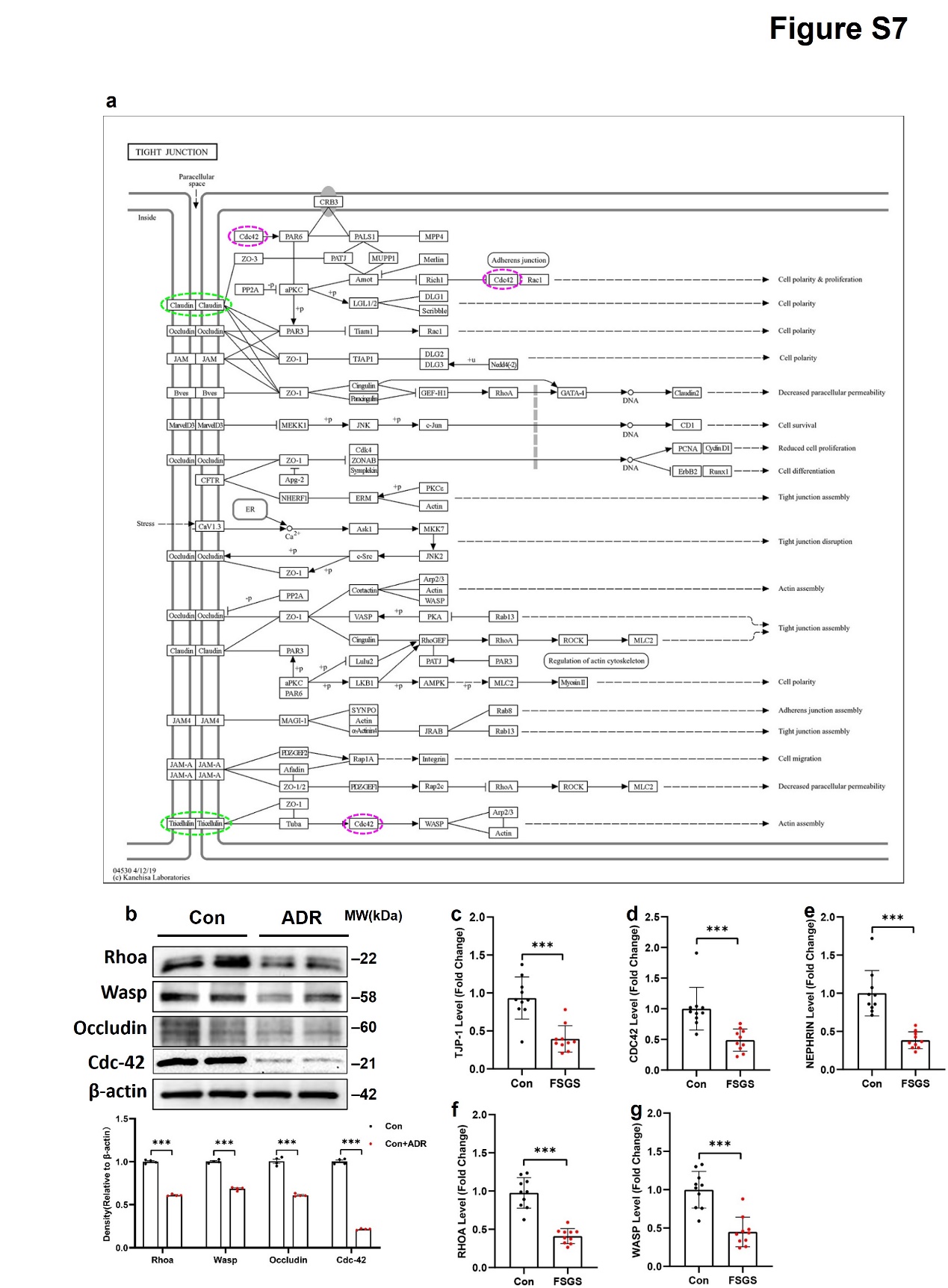


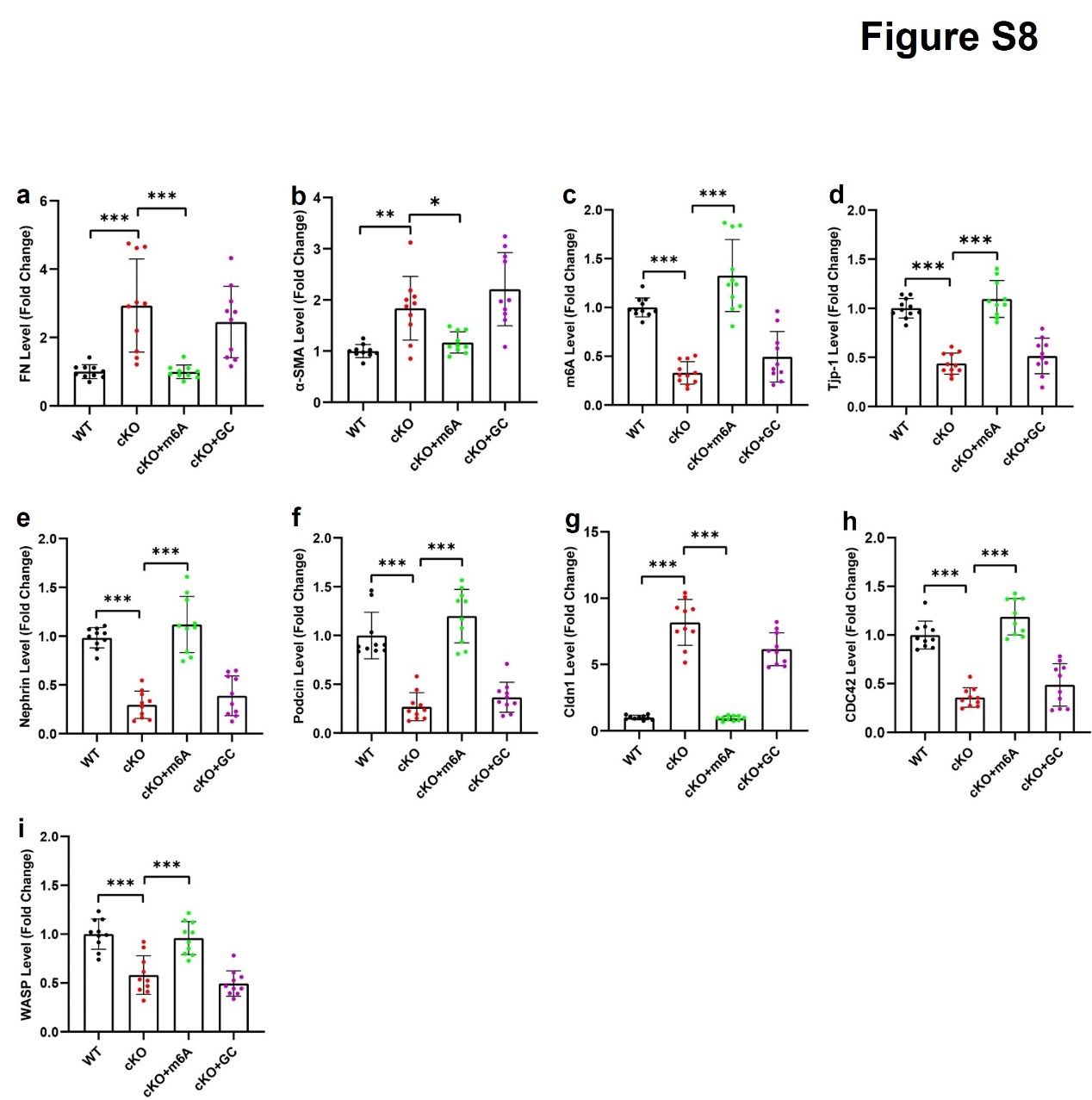


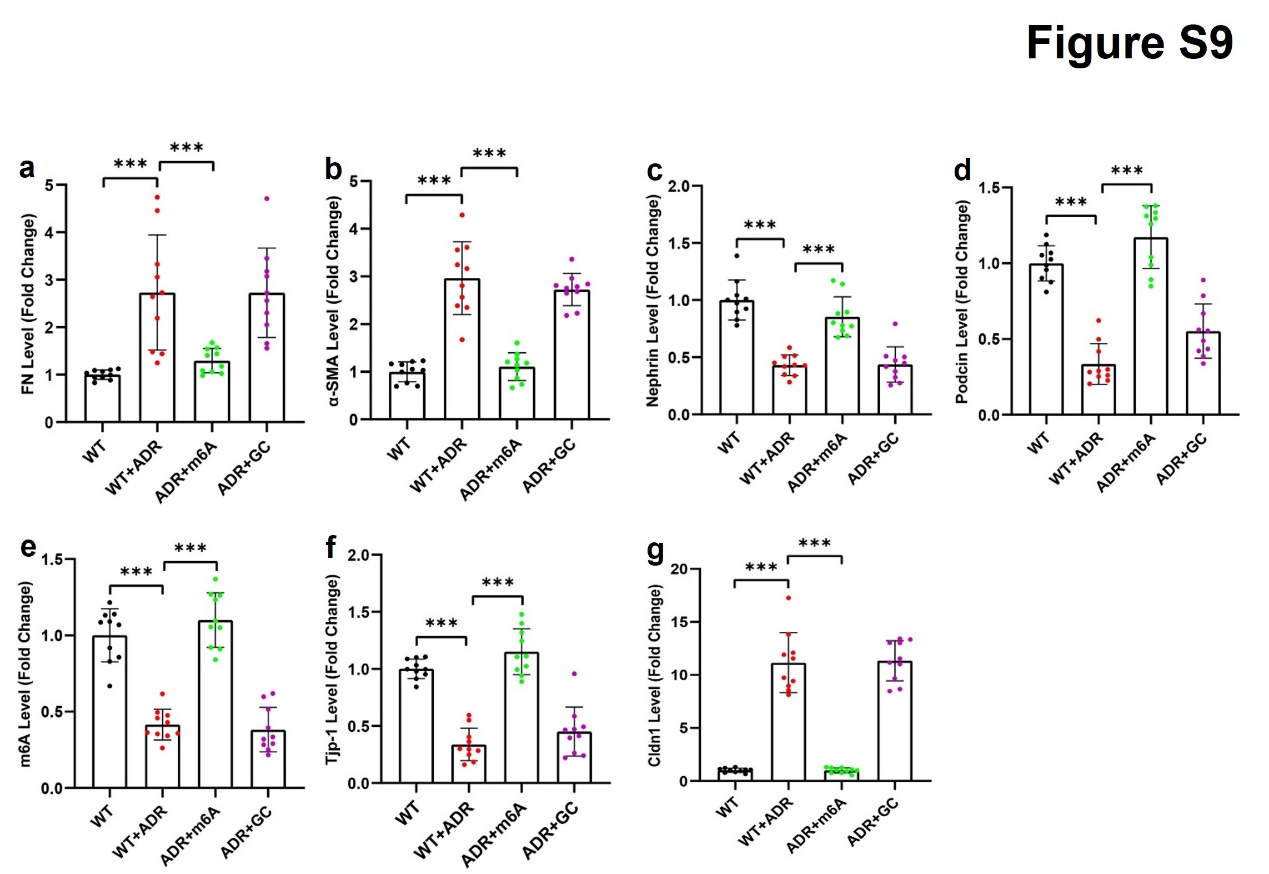


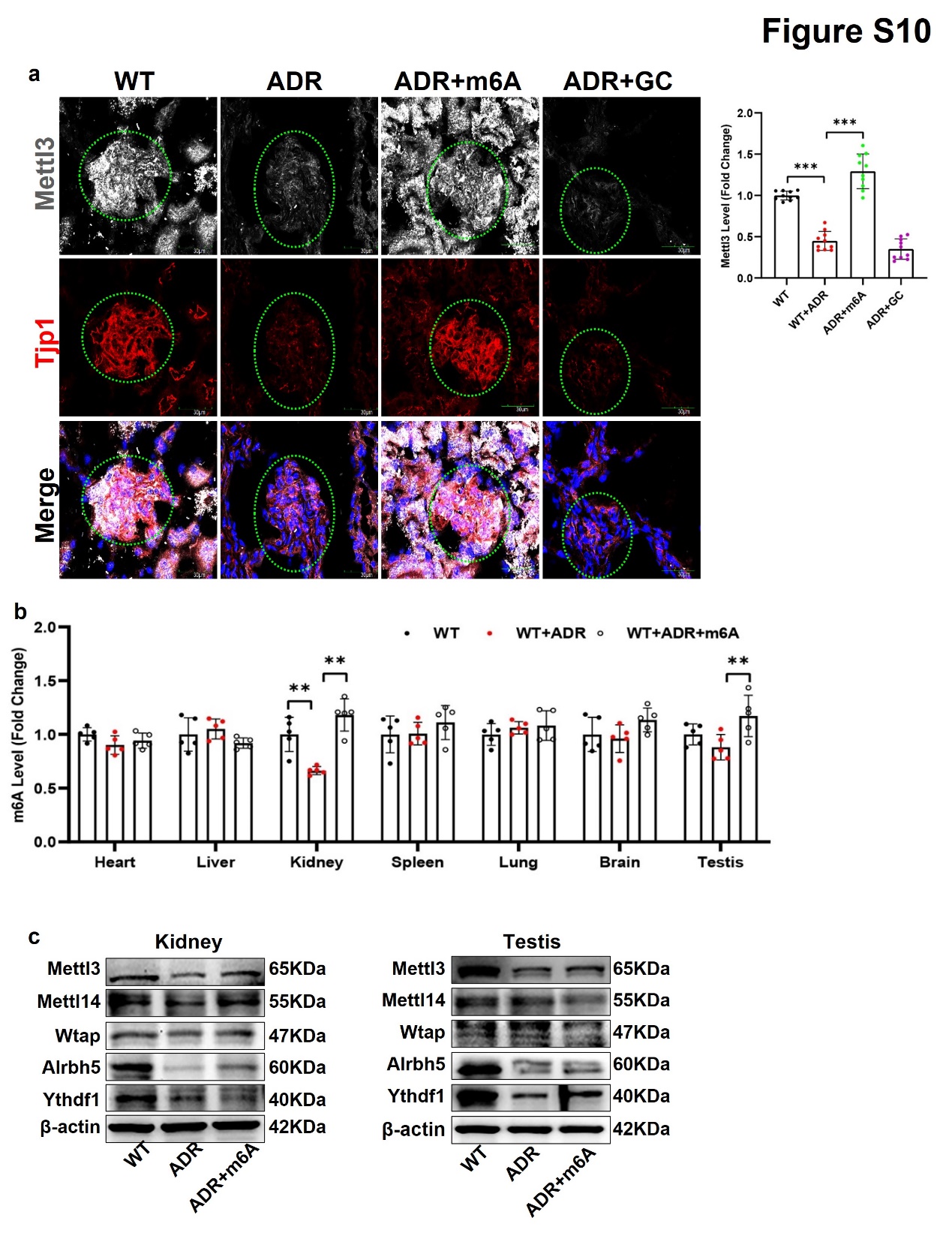


**Supplemental Tables**

**Table S1. Clinical characteristics of FSGS patients**

|  | FSGS(n=14) |
| --- | --- |
| Gender(M/F) | 9/5 |
| Age(Year) | 46.85±5.265 |
| SBP(mmHg) | 133.8±6.51 |
| MBP(mmHg) | 86.67±4.562 |
| Proteinuria(mg/24h) | 9248±1583 |
| Serum Creatinine(uM/L) | 78.9±7.91 |
| eGFR(mL/Min/1.73m2) | 100.3±14.8 |
| Serum Triglyceride(uM/L) | 2.894±0.3375 |
| Serum potassium(mM/L) | 4.04±0.1582 |
| Serum Sodion(mM/L) | 144.5±0.2672 |

**Table S2.** **mRNA primers for q-PCR**

| Gene Name | Forward Primer | Reverse Primer |
| --- | --- | --- |
| msTjp1-1 | CCTGCTAAGCCAGTCCATTC | AAGAGCTGGACAGAGGTGGA |
| msTjp1-2 | ACTCCCACTTCCCCAAAAAC | CCACAGCTGAAGGACTCACA |
| msCldn1 | CTTTCTGCATCTGCCACTGA | ATGTTGTCCCCAGCATAGGA |
| msTmem30b | ACAACACCGGCTTCATCAAC | ACCCATCCAAGAGATGTTGC |
| msMettl3 | CTGGGCACTTGGATTTAAGGAA | TGAGAGGTGGTGTAGCAACTT |
| msMettl14 | CTGAGAGTGCGGATAGCATTG | GAGCAGATGTATCATAGGAAGCC |
| msWtap | TAGACCCAGCGATCAACTTGT | CCTGTTTGGCTATCAGGCGTA |

**Table S3.** **mRNA primers for luciferase assay**

| Gene Name | Forward Primer | Reverse Primer |
| --- | --- | --- |
| Tjp1-1 | ATTCTAGTTGTTTAAACGAGCTCACGACCCTGAAGAGGATGAA | ATGCCTGCAGGTCGACTCTAGAGGACATATATGAGTTCTGA |
| Tjp1-2 | ATTCTAGTTGTTTAAACGAGCTCCTTCTGCCAAGTGAAACTG | ATGCCTGCAGGTCGACTCTAGAGTCTCTGCAGACTTTGAA |

Note: underlines indicate restriction enzyme site, GAGCTC is Sac I site; TCTAGA is Xba I site.

**Table S4. Antibody informations**

| Antibody Name | Company | Catalog Number | Lot Number |
| --- | --- | --- | --- |
| m6A | Abcam | AB208577 | GR260996-5 |
| Mettl3 | Proteintech | 15073-1-AP | 00125922 |
| Nephrin | Santa cruz biotechnology | sc-376522 | E2422 |
| Mettl14 | Bioss | bs-17608R | BB02289435 |
| Wtap | Santa cruz biotechnology | sc-374280 | F2421 |
| Tjp1 | Proteintech | 21773-1-AP | 00127829 |
| Podxl | Bioss | sc-374280 | BC12203966 |
| α-SMA | Abcam | ab5694 | GR3183259-33 |
| Cldn1 | Proteintech | 28674-1-AP | 00150389 |
| Fibronectin | Abcam | ab2413 | GR313897-1 |
| Wasp | Affinity | AF4077 | 88p5704 |
| Alkbh5 | Proteintech | 16837-1-AP | 00161146 |
| Fto | Proteintech | 27226-1-AP | 00152111 |
| Ythdf1 | Proteintech | 17479-1-AP | 00161515 |
| β-actin | Servicebio | GB12001-100 | AC230716005 |

**Table S5. primers for HCR assay**

| Primers | Forward Primer | Reverse Primes |
| --- | --- | --- |
| Tjp1-1 | ACCTTTAGGGAGGTCAAGGA | GAGCCAGTATGGTCACTTCT |
| Tjp1-2 | AGAGCCCAGCTTTCCATTGT | TGCCAATTCGAATGGCAAGC |
| Tjp1-3 | GTACACTGGCTAACTGTTCA | GCTGTCTTTGGAAGTGTGTA |
| Tjp1-4 | TCTCTTCCACCAGAGATTGC | CCCACTCTGAAAATGAGGAT |
| Tjp1-5 | TCCTCTAAGCCTTCTTTGGC | CACCCTGAGAATTTGATCAC |
| Tjp1-6 | TCTTCATCCTCTTCAGGGTC | GGAGAGCTGTTTCCGGTAAT |
| Tjp1-7 | TAACCATTGCAACTCGGTCA | ACGTTATCCATTGAAACTCC |
| Tjp1-8 | TTTCTTCTTCCTTCGGATAG | GGTGACTTACAGGGATCTGA |
| Tjp1-9 | TCGTCTTCATTATCAGACAC | ATGCACTTCTTCGTCATAAC |
| Tjp1-10 | TTCTCGCTCCTTCTGTTCGC | ACTCCTATCCCTTGCCCAGC |
| Tjp1-11 | CCGCTCATCTCTTTGCACTA | CAGGGACGTTCAGTAAGGTA |
| Tjp1-12 | GGCATTAGCAGAATGGATAC | CTGAAATGTCATCTCTTTCC |
| Tjp1-13 | GTTGGTCAGGAGATCGTGAC | GAATGATCGGAGGGCTCTGA |
| Tjp1-14 | CTGGCTGGCTGTACTGTGAG | GAAGAGCTGGACAGAGGTGG |
| Tjp1-15 | GCAACATCAGCTATTGGTCC | CTCTCTTGCCAACTTTTCTC |
| Tjp1-16 | ACTGTAACTTCTTCCACTGC | CGTCTGCTTCTCATTTTTCT |
| Tjp1-17 | CAGGTTTCGGTTCTGGAAGA | GGTTGTCCAACTTGAGCATA |
| Tjp1-18 | GGCTGACGGGTAAATCCACA | TTAGGCAGAGCACCATCAGA |
| Tjp1-19 | TATCCCGTCTTCATGAGCTG | CCAGTTTCATGCTGGGCCTA |
| Tjp1-20 | TCGCAAACCCACACTATCTC | CGACATCATTTCCACCAGCT |
